# Identification of a short, highly conserved, motif required for picornavirus capsid precursor processing at distal sites

**DOI:** 10.1371/journal.ppat.1007509

**Published:** 2019-01-18

**Authors:** Thea Kristensen, Graham J. Belsham

**Affiliations:** DTU National Veterinary Institute, Technical University of Denmark, Lindholm, Kalvehave, Denmark; University of California, Irvine, UNITED STATES

## Abstract

Many picornaviruses cause important diseases in humans and other animals including poliovirus, rhinoviruses (causing the common cold) and foot-and-mouth disease virus (FMDV). These small, non-enveloped viruses comprise a positive-stranded RNA genome (ca. 7–9 kb) enclosed within a protein shell composed of 60 copies of three or four different capsid proteins. For the aphthoviruses (e.g. FMDV) and cardioviruses, the capsid precursor, P1-2A, is cleaved by the 3C protease (3C^pro^) to generate VP0, VP3 and VP1 plus 2A. For enteroviruses, e.g. poliovirus, the capsid precursor is P1 alone, which is cleaved by the 3CD protease to generate just VP0, VP3 and VP1. The sequences required for correct processing of the FMDV capsid protein precursor in mammalian cells were analyzed. Truncation of the P1-2A precursor from its C-terminus showed that loss of the 2A peptide (18 residues long) and 27 residues from the C-terminus of VP1 (211 residues long) resulted in a precursor that cannot be processed by 3C^pro^ although it still contained two unmodified internal cleavage sites (VP0/VP3 and VP3/VP1 junctions). Furthermore, introduction of small deletions within P1-2A identified residues 185–190 within VP1 as being required for 3C^pro^-mediated processing and for optimal accumulation of the precursor. Within this C-terminal region of VP1, five of these residues (YCPRP), are very highly conserved in all FMDVs and are also conserved amongst other picornaviruses. Mutant FMDV P1-2A precursors with single amino acid substitutions within this motif were highly resistant to cleavage at internal junctions. Such substitutions also abrogated virus infectivity. These results can explain earlier observations that loss of the C-terminus (including the conserved motif) from the poliovirus capsid precursor conferred resistance to processing. Thus, this motif seems essential for maintaining the correct structure of picornavirus capsid precursors prior to processing and subsequent capsid assembly; it may represent a site that interacts with cellular chaperones.

## Author summary

The picornavirus family includes clinically important human and animal pathogens, for example: poliovirus, rhinovirus (causing the common cold) and foot-and-mouth disease virus (FMDV) that infects cloven-hoofed animals. Picornaviruses contain a positive-sense RNA genome surrounded by a protein shell, also called a capsid. The capsid proteins are made from a precursor and correct processing and assembly of these capsid proteins is necessary in the virus life cycle to create new infectious virus particles. In this study, we have identified a short motif (just 5 amino acids long) within the capsid precursor, which is highly conserved among picornaviruses. Deletion of this motif inhibited processing of the junctions between the mature structural proteins within this precursor, with one junction being more than 400 amino acids away from this region. This motif also seems to be required for the optimal accumulation of the capsid precursor in cells. We hypothesize that the motif may be involved in binding to a cellular protein, such as a chaperone, to stabilize the capsid precursor and promote its correct folding to allow it to be processed by the viral protease prior to capsid assembly.

## Introduction

Picornaviruses comprise a large family of non-enveloped RNA viruses that includes important human and animal pathogens. Examples include poliovirus (PV) (genus: *Enterovirus*), hepatitis A virus (*Hepatovirus*), encephalomyocarditis virus (*Cardiovirus*) and foot-and-mouth disease virus (FMDV) *(Aphthovirus)*.

In picornavirus particles, the RNA genome (ca. 7,100–8,900 nt) is surrounded by a protein shell (capsid) consisting of the four structural proteins VP1, VP2, VP3 and VP4 [1], with the exception of parechoviruses and kobuviruses in which the VP0 (the precursor of VP2 and VP4) remains uncleaved (reviewed by [2]). The capsid is composed of 60 copies of each of these structural proteins; VP1, VP2 and VP3 are exposed on the surface of the particle while VP4 is entirely internal [3–5].

**Fig 1:**
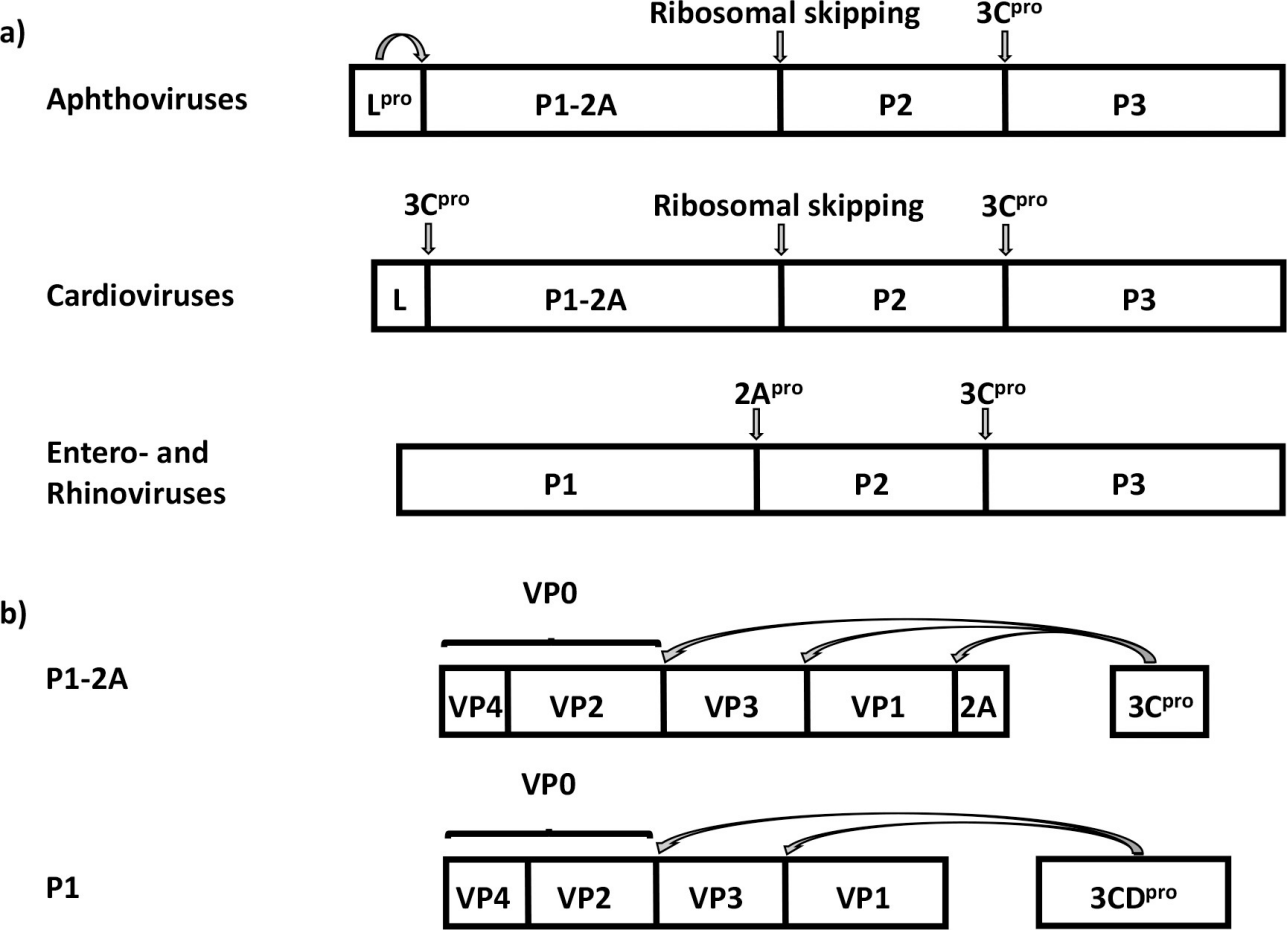
Schematic representation of the polyprotein processing in three different genera of picornaviruses. a) The RNA genome of viruses in the genus *Aphthovirus*, in which FMDV is the prototypic member, encodes a polyprotein that is processed into four primary products namely the Leader protease (L^pro^), P1-2A, P2 and P3 during, and after, synthesis. The “cleavage” at the 2A/2B junction occurs during synthesis by a mechanism known as “ribosomal skipping” [8] or “StopGo” [9]. The L^pro^ mediated cleavage occurs at its own C-terminus, i.e. at the L/P1-2A junction while the 3C^pro^ is responsible for cleavage of the P2/P3 junction. The *Cardiovirus* polyprotein is also processed into the primary products, Leader, P1-2A, P2 and P3 but the Leader protein is removed from the capsid precursor by the 3C^pro^. The *Entero*- and *Rhinoviruses*, do not have a Leader protein and the polyprotein is thus processed into only three primary products, namely P1, P2 and P3. In the *Entero*- and *Rhinoviruses* the 2A protein is a protease that cleaves the P1/P2 junction. The 3C^pro^ is responsible for cleavage of the P2/P3 junction. b) The 3C^pro^ is sufficient for processing of the capsid precursor, P1-2A, in *Aphthoviruses* and *Cardioviruses*. The precursor is processed to generate VP0, VP3 and VP1, and a short 2A peptide. In *Entero*- and *Rhinoviruses* the 3CD^pro^ is responsible for processing the capsid precursor P1 to VP0, VP3 and VP1.

Translation of the positive-sense RNA genome is dependent on the internal ribosomal entry site (IRES) within the 5**′** untranslated region (UTR) that directs cap-independent translation initiation [6]. During and after translation of the single open reading frame, processing of the newly synthesized polyprotein occurs (reviewed in [2]). Usually three or four primary products are formed, namely the Leader (in many picornaviruses), the capsid precursor P1 or P1-2A (depending on the genus) and the precursors of the non-structural proteins, namely P2 and P3. Many of these viruses, e.g. members of the *Cardiovirus*, *Hepatovirus* and *Aphthovirus* genera, have a Leader protein at the N-terminus of the polyprotein, i.e. upstream of the capsid precursor. In the *Aphthoviruses*, the Leader protein is a protease (L^pro^), which cleaves itself from the N-terminus of the P1-2A precursor, see Fig 1. Cleavage of the junction between the structural and non-structural proteins, at either the VP1/2A or the 2A/2B junction, is usually mediated by the 2A protein, but the function of the 2A protein varies between the genera [7], see Fig 1A. In the cardio- and aphthoviruses cleavage at the 2A/2B junction (at the C-terminus of 2A) is protease independent and happens during translation by a process termed “ribosomal skipping” [8] or “StopGo” [9]. In this case, the 2A protein remains attached to the precursor of the structural proteins (as P1-2A) until it is removed by the 3C protease (3C^pro^), see Fig 1B. In the enteroviruses, the cleavage at the VP1/2A junction (i.e. at the N-terminus of 2A), to release P1, is mediated by the 2A protein that is a chymotrypsin-like protease [10,11].

The P2-P3 junction and the other protein junctions within these precursors are cleaved by 3C^pro^ to produce the mature non-structural proteins. However, the P1 capsid precursor of enteroviruses requires the 3CD protease (3CD^pro^) for its processing [12,13] whereas for the cardio- and aphthoviruses the 3C^pro^ is sufficient to cleave the P1-2A precursor into three structural proteins (VP0, VP3 and VP1) plus 2A [1,14], see Fig 1B. During capsid assembly, VP0 is cleaved (in most picornaviruses) to generate VP2 and VP4 by a process that is currently not understood.

There are seven different serotypes of FMDV: O, A, C, SAT 1, SAT 2, SAT 3 and Asia 1. There is a high level of sequence variation between the surface exposed structural proteins of these different serotypes. The internal VP4 protein is the most conserved of the capsid proteins with 81% of the residues being invariant [15]. In contrast, only 26% of the VP1 protein residues are invariant and furthermore it ranges in size (209–213 aa) between serotypes [16]. VP1 is the most surface exposed capsid protein [3] and has been one of the most studied FMDV proteins due to its antigenic importance and role in virus attachment [17].

One of the antigenic sites in VP1 is located on the G-H loop (including residues 141–160), which contains an arginine-glycine-aspartate (RGD) motif that is involved in the attachment of the virus to cellular integrin receptors [18,19]. Surprisingly, previous work has demonstrated that a cell-culture adapted FMDV, lacking part of this G-H loop (aa 142–154), is still able to replicate and grow normally in cell culture through the use of heparan sulfate proteoglycans (HSPG) as receptor [20].

Viruses have only a very limited coding capacity within their genomes and thus they rely on cellular factors and pathways to complete their life cycle. Several studies have suggested that cellular chaperones, including various different heat shock proteins (Hsps), are required to facilitate virus entry, genome replication, protein expression and protein assembly for a variety of viruses, including picornaviruses. Viral proteins, like cellular proteins, are dependent on such chaperones for their correct folding and assembly [21–24]. Studies on the role of Hsp90, using specific inhibitors, have shown that these agents reduce the replication of diverse viruses *in vitro*. The Hsp90 appears to be involved in the regulation of viral polymerase function in the case of herpesvirus [25] and hepatitis B virus [21], whereas this chaperone seems to be required for capsid processing and assembly in different picornaviruses [23,26]. Hsp90 and Hsp70 have been reported to interact with the PV capsid precursor, P1 [23,27]. The interaction between PV P1 and Hsp90 (possibly together with Hsp70), and likely in conjunction with its co-chaperone p23, is believed to protect the P1 from degradation by proteasomes (which remove misfolded proteins) and is also involved in the folding of P1 allowing it to be correctly processed by the 3CD^pro^ [23].

Recently, we have shown that impeding the processing of one of the cleavage sites within the FMDV P1-2A, at either the VP0-VP3 or the VP3-VP1 junctions, did not block processing of the other cleavage sites, indicating that processing of these junctions is mutually independent [28]. However, in an earlier study, it was shown that truncation of VP1 (removing the C-terminal 42 amino acids of VP1) completely blocked processing of the residual capsid precursor at both the VP0-VP3 and the VP3-VP1 junctions by 3C^pro^ in a cell-free system [29]. Similarly, truncating the PV P1 precursor, by removing 50 aa from the C-terminus of VP1 (302 residues in length), blocked cleavage of the 2 junctions within the P1 precursor *in vitro* [30]. The basis for these effects has not been explained. However, taken together, these results suggest that the C-terminus of VP1 is important in relation to the processing of the entire capsid precursor of picornaviruses.

In this study, we have now identified a short region within the C-terminus of VP1 that is critical for the processing of the FMDV capsid precursor. This region contains a stretch of five amino acids that are very highly conserved amongst all FMDVs. Furthermore, this region is also strongly conserved between most other picornaviruses, including PV, suggesting a shared role for this motif for capsid processing and assembly within the picornavirus family.

## Results

### Truncation of the capsid precursor prevents its processing by 3C^pro^ in cells

**Fig 2:**
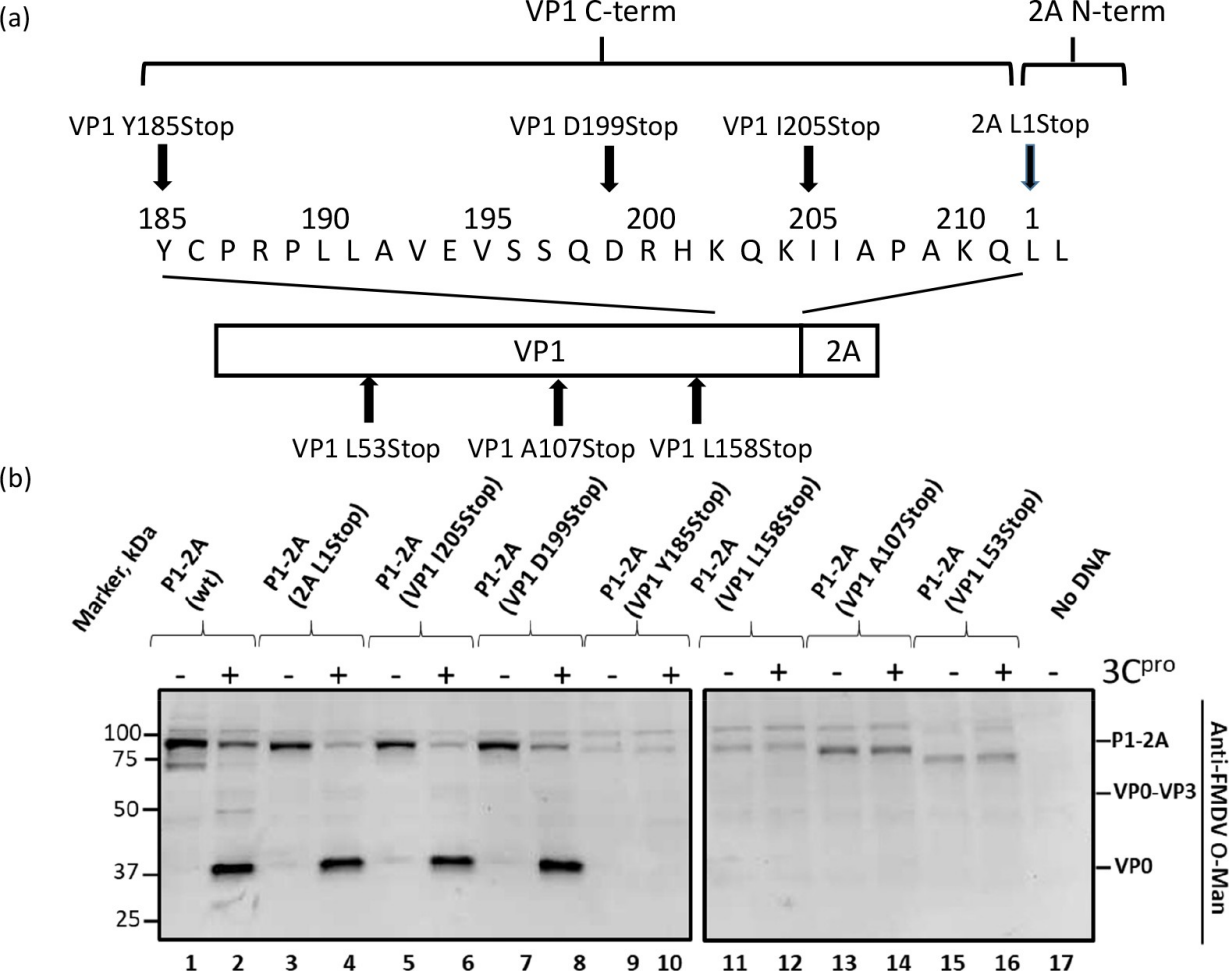
Truncations in the C-terminus of FMDV VP1 affect capsid precursor processing. a) Constructs encoding the FMDV P1-2A (wt) were modified to introduce truncations at different sites in the VP1 coding sequence. The 2A L1Stop truncation served as a second positive control for processing. b) Cell lysates from BHK cells (infected with vTF7-3 [45]) transfected with plasmids that express the FMDV P1-2A (wt or truncated at the VP1) alone (odd numbered lanes) or with the plasmid pSKRH3C [46] encoding the FMDV 3C^pro^ (even numbered lanes) were analyzed by immunoblotting. Molecular mass markers (kDa) are indicated on the left. A negative control (No DNA) was included (lane 17). The different structural proteins are indicated on the right of the figure and were detected using guinea pig anti-FMDV O-Man antisera. Bound antibodies were visualized using the anti-guinea pig HRP-conjugated secondary antibodies and a chemiluminescence detection kit.

Previous studies have shown that truncation of the FMDV P1-2A, by removal of the 2A peptide and the C-terminal 42 residues of VP1, completely abrogated processing by 3C^pro^ *in vitro* [29] even though the cleavage sites between VP0 and VP3 and between VP3 and VP1 were unmodified. To confirm these observations, within cells, stop codons were introduced at different positions within the P1-2A coding sequence. Transient expression assays were used to express the FMDV A22 Iraq P1-2A capsid precursor and its derivatives, within BHK cells, both in the absence and presence of the FMDV 3C^pro^. The plasmids encoding both the P1-2A (wt) and the P1 alone (truncated to the first amino acid of the 2A peptide) served as positive controls. Both of these controls yielded the expected products corresponding to the P1-2A precursor and the P1 precursor (approximately 85 kDa), respectively in the absence of 3C^pro^ (Fig 2, lanes 1 and 3). When these plasmids were co-transfected with a plasmid that expresses the 3C^pro^, both of these products were efficiently processed as indicated by the production of VP0 (approximately 37 kDa) (Fig 2, lanes 2 and 4). Thus, the absence of the 2A peptide did not affect processing of the capsid precursor by 3C^pro^ (as observed previously [14,26]).

Plasmids encoding mutant precursors, truncated to residue 205 in VP1 and 199 in VP1 (VP1 being 211 aa in length in FMDV A22 Iraq (wt)), generated products of approximately 85 kDa in the absence of 3C^pro^ (Fig 2, lanes 5 and 7), and these were efficiently processed in the presence of 3C^pro^ (Fig 2, lanes 6 and 8). The four additional mutants, P1 (VP1 Y185Stop), P1 (VP1 L158Stop), P1 (VP1 A107Stop) and P1 (VP1 L53Stop) all yielded products corresponding to their expected size in the absence of 3C^pro^ (Fig 2, lanes 9, 11, 13 and 15), however it is noteworthy that these truncated products accumulated to a lower level in the cell lysates. Strikingly, no processing of these truncated precursors was detected for any of these four mutants in the presence of 3C^pro^ (Fig 2, lanes 10, 12, 14 and 16) although each of these products contained the unmodified VP0/VP3 and VP3/VP1 junctions. As expected, no products were detected in the negative control (no DNA) (Fig 2, lane 17).

### Mapping of the determinants of 3C^pro^ mediated processing of the capsid-precursor

**Fig 3:**
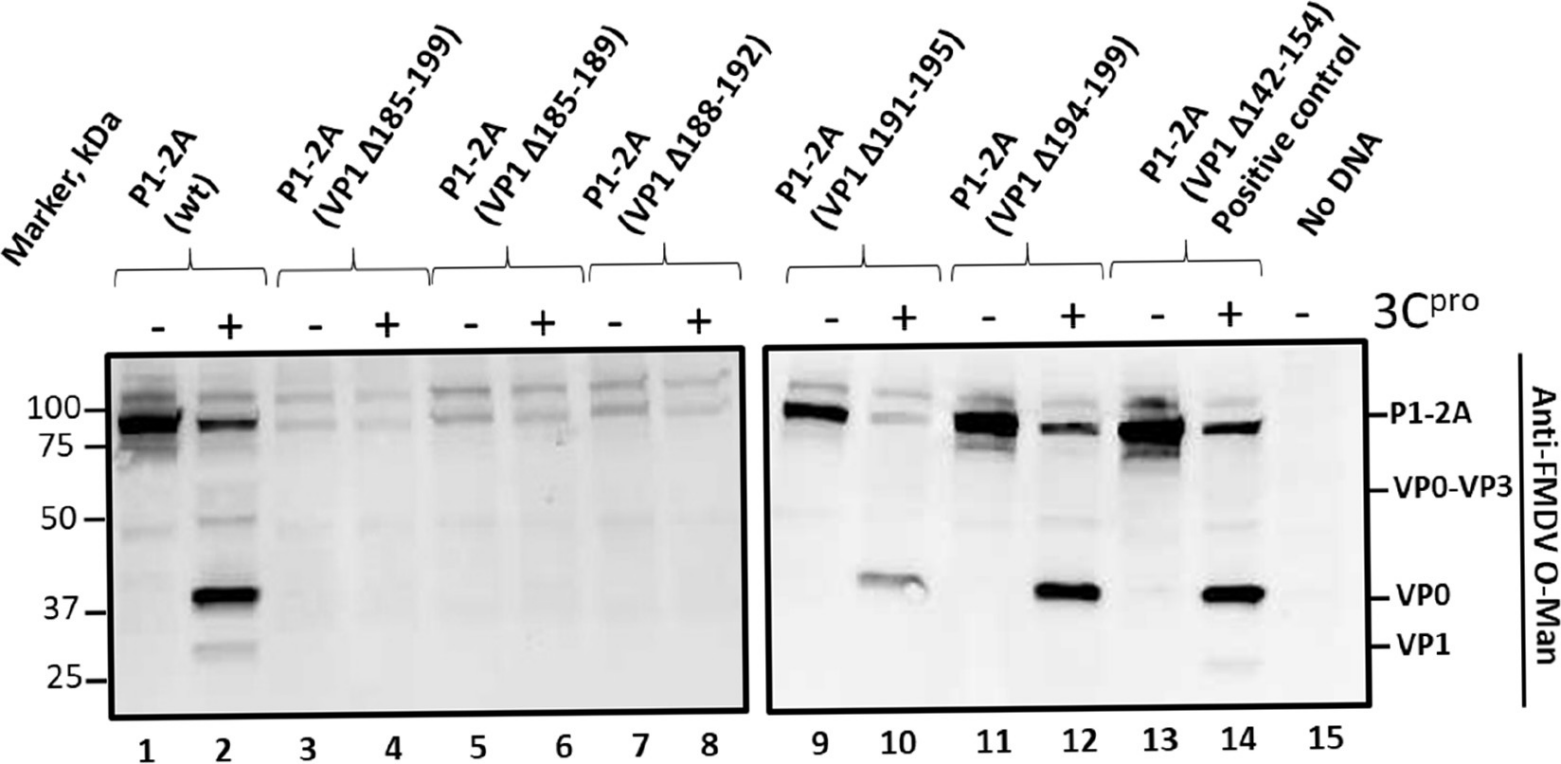
A short region near the C-terminus of FMDV VP1 is required for capsid precursor processing Cell lysates were prepared from BHK cells (infected with vTF7-3 [45]) following transfection with plasmids that express the FMDV P1-2A wt or with small deletions as indicated either alone (odd numbered lanes) or with the plasmid pSKRH3C [46] encoding the 3C^pro^ (even numbered lanes). The samples were analyzed by immunoblotting. A positive control with a deletion known to be tolerated in infectious FMDV [20] was included, P1-2A (VP1 Δ142–154), lanes 13 and 14. Molecular mass markers (kDa) are indicated on the left. A negative control (No DNA) was used in lane 15. The structural proteins corresponding to the different bands are indicated on the right of the figure and were detected using guinea pig anti-FMDV O-Man antisera. Bound antibodies were visualized using the anti-guinea pig HRP-conjugated secondary antibodies and a chemiluminescence detection kit.

In order to map the determinants of capsid processing more precisely, plasmids were constructed to express mutant forms of the P1-2A precursor with fairly small internal deletions within the C-terminal portion of VP1. To serve as positive controls, both the P1-2A (wt) and a mutant form with a deletion within VP1, designated P1-2A (VP1 Δ142–154), were included. The latter deletion is tolerated by the infectious virus [20] and thus it was expected that 3C^pro^ should be able to fully process all of the junctions in this deletion mutant. As expected, expression of both the P1-2A (wt) and the P1-2A (VP1 Δ142–154) led to the synthesis of products corresponding to the P1-2A precursor (approximately 85 kDa) (Fig 3, lanes 1 and 13). Furthermore, both the P1-2A (wt) and the P1-2A (VP1 Δ142–154) products were efficiently processed in the presence of 3C^pro^ (Fig 3, lanes 2 and 14). Notice that the VP1 product derived from the P1-2A (VP1 Δ142–154) mutant migrated faster than the VP1 produced from the P1-2A (wt) ((approximately 28 kDa) due to the internal deletion (note that these antibodies do not recognize VP3 [31], but presumably this was also made).

**Fig 4:**
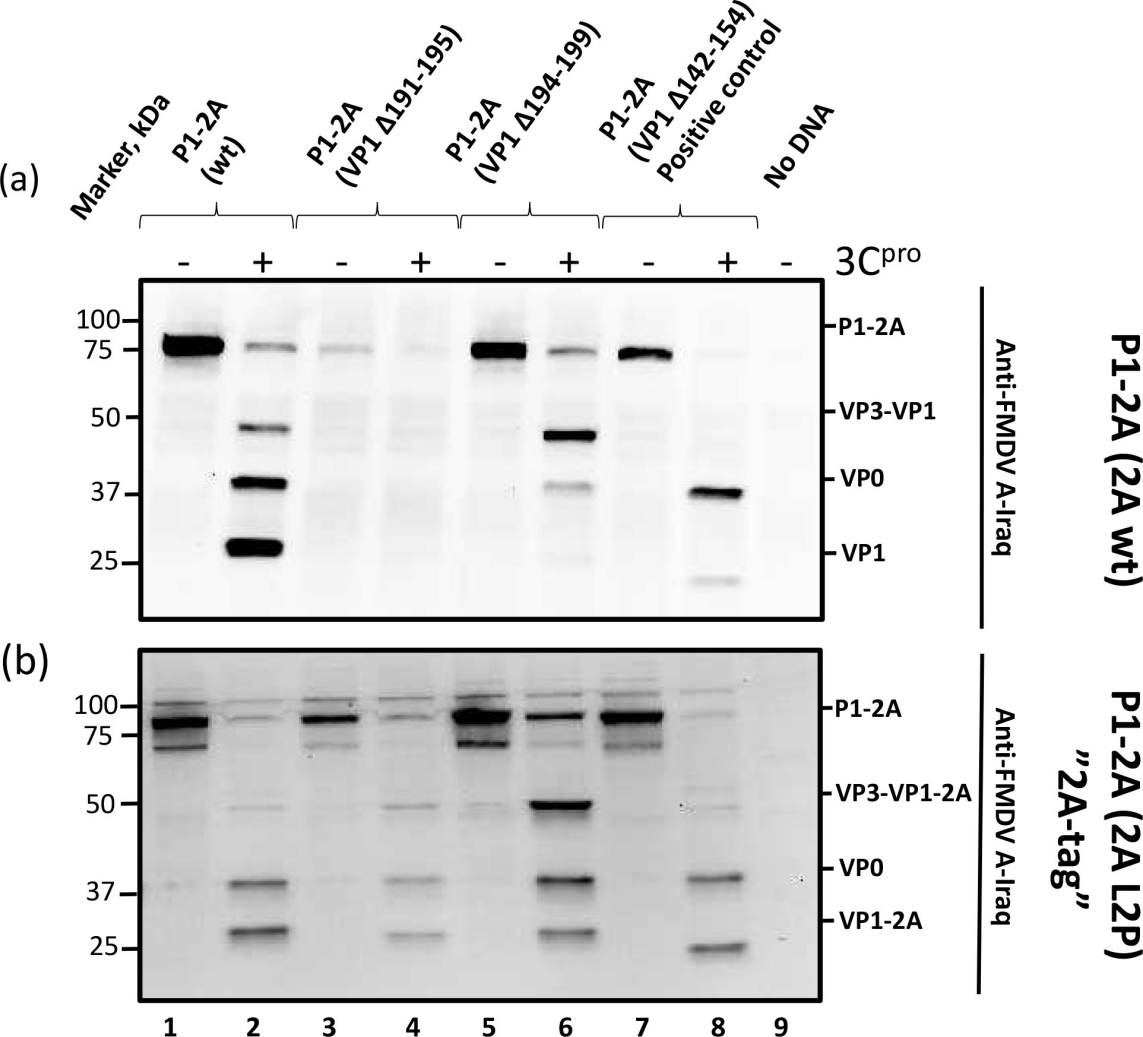
A region near the C-terminus of FMDV VP1 required for processing of the VP3/VP1 junction. a) The P1-2A precursor (wt or with small deletions as indicated) was expressed alone or with 3C^pro^ in transient expression assays in BHK cells as for Fig 3. Cell lysates were prepared and analyzed by immunoblotting. The structural proteins corresponding to the different bands are indicated and were detected using guinea pig anti-FMDV A-Iraq antisera. Bound antibodies were visualized using the anti-guinea pig HRP-conjugated secondary antibodies and chemiluminescence detection. The odd numbered lanes show the P1-2A precursors expressed alone and the even numbered lanes show the precursor co-expressed with 3C^pro^. Molecular mass markers (kDa) are indicated on the left. A negative control (No DNA) is included in lane 9. A positive control with a deletion known to be tolerated in replicating FMDV [20] was included, P1-2A (VP1 Δ142–154), lanes 7 and 8. b) The 2A L2P substitution was introduced into the plasmids encoding P1-2A (wt), P1-2A (VP1 Δ191–195), P1-2A (VP1 Δ194–199) and the positive control P1-2A (VP1 Δ142–154). This 2A L2P substitution blocks cleavage of the VP1/2A junction and increased the sensitivity of detection of VP1. The negative control (No DNA) is indicated.

Five different short deletions were introduced into the region of VP1 spanning residues 185–199 (the region found to be critical by the truncation analysis), namely P1-2A (VP1 Δ185–199), P1-2A (VP1 Δ185–189), P1-2A (VP1 Δ188–192), P1-2A (VP1 Δ191–195) and P1-2A (VP1 Δ194–199). Each of these constructs generated products that were very similar in size as the wt P1-2A in the absence of 3C^pro^ (Fig 3, lanes 3, 5, 7, 9 and 11). However, in the presence of 3C^pro^ the mutant having the largest deletion, P1-2A (VP1 Δ185–199) could not be processed (Fig 3, lane 4). The same product, corresponding to the P1-2A precursor, was observed both in the absence and presence of 3C^pro^. Similarly, the mutants P1-2A (VP1 Δ185–189) and P1-2A (VP1 Δ188–192) were also not processed in the presence of 3C^pro^ (Fig 3, lanes 6 and 8). It is again noteworthy that the mutant P1-2A products that could not be processed accumulated to a lower level in the cell lysates than the P1-2A precursors that could be processed (c.f. lanes 3, 5, 7 and 1, 9, 11, 13). In contrast, co-expression of 3C^pro^ with the P1-2A (VP1 Δ191–195) and P1-2A (VP1 Δ194–199) led to production of VP0 indicating that processing of these mutant precursors had occurred (Fig 3, lanes 10 and 12). However, it is noteworthy that no product corresponding to VP1 was detected, when P1-2A (VP1 Δ191–195) was co-expressed with 3C^pro^ (Fig 4A, lane 4). Furthermore, unexpectedly, when the P1-2A (VP1 Δ194–199) was co-expressed with 3C^pro^ a major product corresponding to the intermediate VP3-VP1 (approximately 49 kDa) was detected (Fig 4A, lane 6). Only a weak signal corresponding to the mature VP1 was detected indicating severe inhibition of processing at the VP3/VP1 junction in this mutant (Fig 4A, lane 6), n.b. this cleavage site is located over 190 residues away in the linear sequence. No products were detected in the negative control lane. (Fig 4A, lane 9).

Due to inefficient detection of VP1 from some of the mutant precursors, an extra modification that blocks processing of the VP1/2A junction (2A L2P) [32] was introduced into the plasmids that express P1-2A (VP1 Δ191–195), P1-2A (VP1 Δ194–199) and the positive controls; P1-2A (wt) and P1-2A (VP1 Δ142–154). The additional modification (2A L2P) ensured that the 2A peptide remained fused to the VP1 (as VP1-2A). Each of these constructs generated products corresponding to the P1-2A precursor in the absence of 3C^pro^ (Fig 4B, lanes 1, 3, 5 and 7). The 2A L2P substitution increased the sensitivity of VP1 detection when using the anti-FMDV A-Iraq antibody. This showed that the P1-2A (wt + 2A L2P) and the P1-2A (VP1 Δ142–154 + 2A L2P, positive control) precursors were fully processed to yield VP0 and VP1-2A in the presence of 3C^pro^ as expected (Fig 4B, lanes 2 and 8). It also verified that cleavage at the VP3-VP1 junction in the P1-2A (VP1 Δ194–199 +2A L2P) occurred at a slower rate compared to wt, since the VP3-VP1-2A intermediate was far more abundant for the P1-2A (VP1 Δ194–199 + 2A L2P) than for the P1-2A (wt +2A L2P) in the presence of 3C^pro^ (compare lanes 6 and 2 in Fig 4B). It should be noted that some mature VP1-2A could be detected from the P1-2A (VP1 Δ194–199 + 2A L2P) and thus cleavage of the VP3/VP1 junction was not completely blocked (Fig 4B, lane 6). Furthermore, the P1-2A (VP1 Δ191–195 + 2A L2P) could be processed to generate VP0 and VP1-2A (Fig 4B, lane 4). However, the VP3-VP1 intermediate produced from the P1-2A (VP1 Δ191–195 +2A L2P) mutant was also more abundant than the intermediate seen with the P1-2A (wt + 2A L2P) indicating that this mutant also had a slower processing at the VP3/VP1 junction (Fig 4B, lane 4).

The cleavage of the unmodified VP1/2A junction in the P1-2A precursors with different internal deletions, was investigated using an anti-2A antibody. As expected, both the P1-2A (wt) and the positive control P1-2A (VP1 Δ142–154) generated products of approximately 85 kDa corresponding to the P1-2A precursor in the absence of 3C^pro^ (see supplementary material S1 Fig, lanes 1 and 13). In the presence of 3C^pro^, no products were detected by the anti-2A antibodies from either the P1-2A (wt) or the positive control P1-2A (VP1 Δ142–154) indicating that the VP1/2A junction had been processed (S1 Fig, lanes 2 and 14); note the 2A peptide itself is only 18 residues long and is not detected by immunoblotting. The two mutants, P1-2A (VP1 Δ191–195) and P1-2A (VP1 Δ194–199) that showed slower processing of the VP3-VP1 junction also generated products corresponding to the P1-2A precursor in the absence of 3C^pro^ (S1 Fig, lanes 9 and 11). However, in the presence of 3C^pro^, no products were detected by the anti-2A antibodies (S1 Fig, lanes 10 and 12), indicating that these two deletions in VP1 did not affect processing of the VP1/2A junction. Surprisingly, the non-processable precursors, i.e. P1-2A (VP1 Δ185–199), P1-2A (VP1 Δ185–189) and P1-2A (VP1 Δ188–192), could not be detected using the anti-2A antibody, either in the absence or presence of 3C^pro^, and thus we cannot conclude whether cleavage of this junction was affected by the deletions (S1 Fig, lanes 3–8). No products were detected in the negative control (No DNA, S1 Fig, lane 15).

### Identification of critical residues in the P1-2A-precursor for 3C^pro^ processing

**Fig 5:**
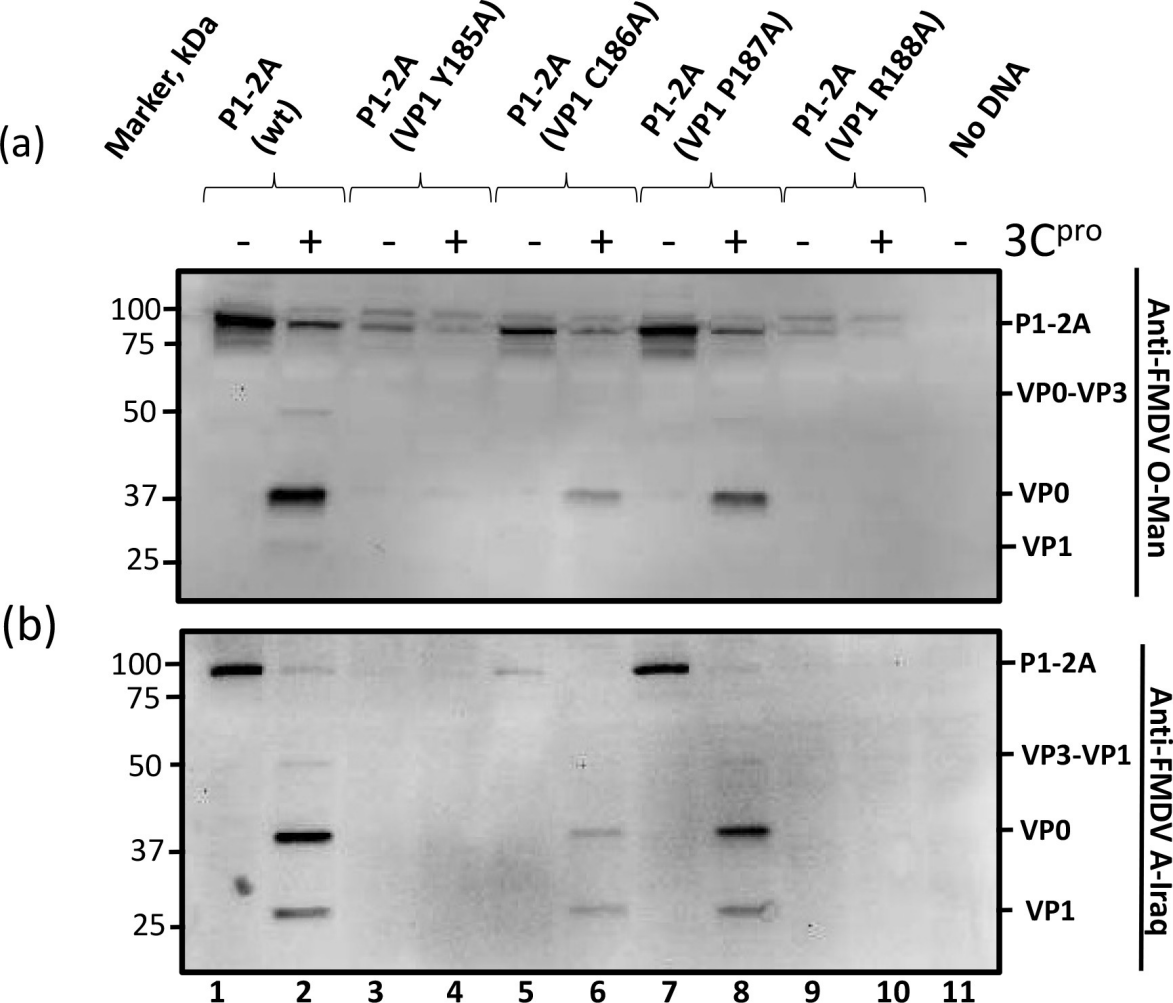
Identification of amino acids between residues VP1 185 and 188 required for capsid precursor processing The P1-2A precursors (wt or having alanine substitutions between VP1 185 and VP1 188) were expressed alone or in the presence of 3C^pro^ and analyzed by immunoblotting (as above). The structural proteins corresponding to the different products are indicated on the right of the figure and were detected using guinea pig anti-FMDV O-Man antisera (a) or guinea pig anti-FMDV A-Iraq antisera (b). Bound antibodies were visualized using the anti-guinea pig HRP-conjugated secondary antibodies and a chemiluminescence detection kit. Molecular mass markers (kDa) are indicated on the left. A negative control (No DNA) is included in lane 11.

**Fig 6:**
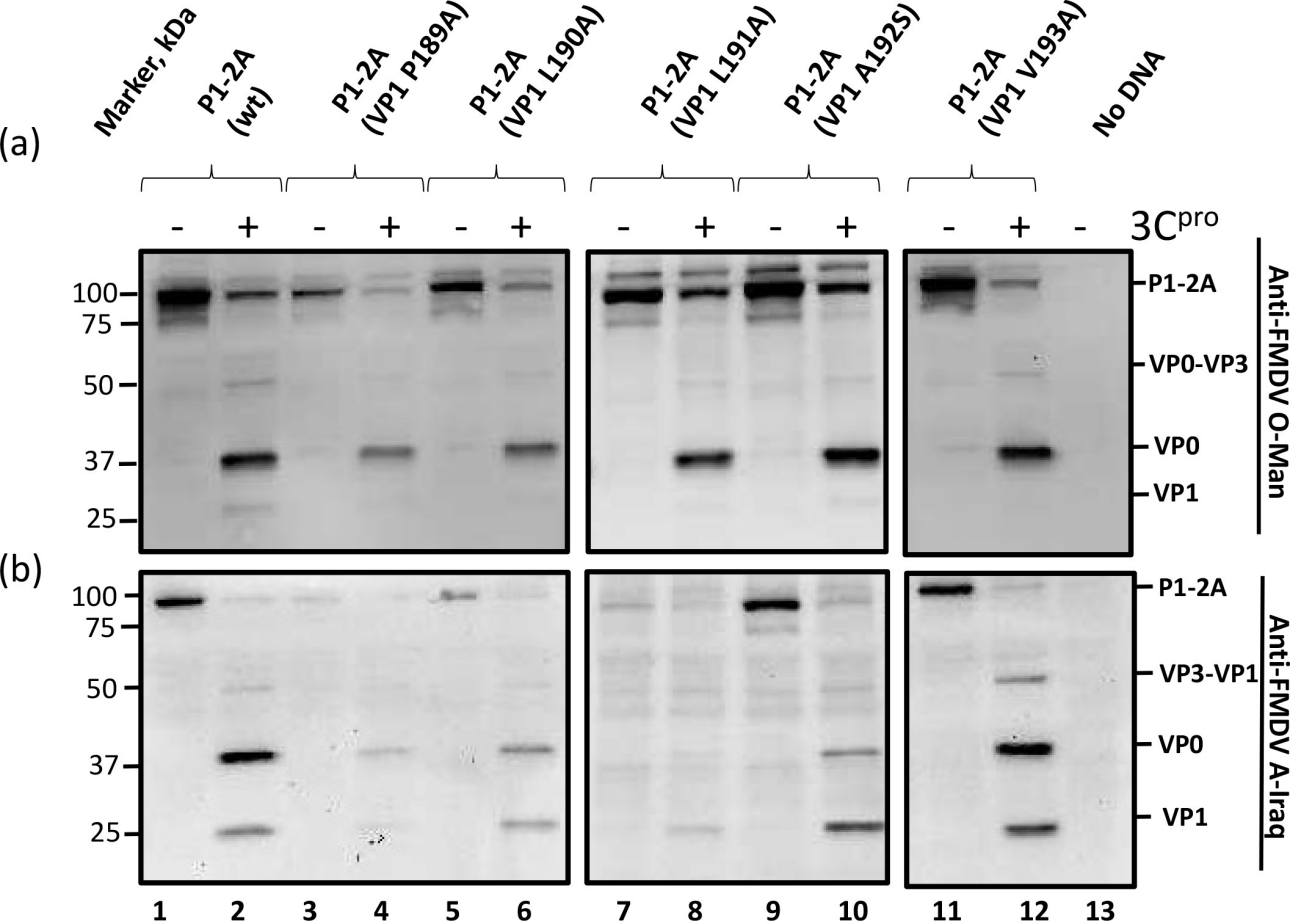
Identification of residues between VP1 189 and 193 required for processing at the VP3/VP1 junction. The P1-2A precursors (wt or with alanine substitutions between VP1 189 and VP1 193, with the exception of VP1 192 where an alanine were substituted to a serine, as indicated) were expressed alone or in the presence of 3C^pro^ and analyzed by immunoblotting. The capsid proteins are indicated on the right of the figure. Proteins were detected using guinea pig anti-FMDV O-Man antisera (a) or with guinea pig anti-FMDV A-Iraq antisera (b). Bound antibodies were visualized using the anti-guinea pig HRP-conjugated secondary antibodies and a chemiluminescence detection kit. Molecular mass markers (kDa) are indicated on the left. A negative control (No DNA) is included in lane 13.

Alanine-scanning mutagenesis was employed to identify individual residues within the C-terminal region of VP1 (between residues 185 and 199 of VP1) that are required for 3C^pro^ processing of the P1-2A precursor. The wt and mutant precursors were expressed alone and also in the presence of the FMDV 3C^pro^ as above. As expected, the P1-2A (wt) and all 15 of the single amino acid substitution mutants each generated products corresponding to the P1-2A precursor in the absence of 3C^pro^ (see Figs 5, 6 and S2 (supplementary material), odd numbered lanes). The wt and some 13 different mutant P1-2A precursors, excluding the mutants P1-2A (VP1 Y185A) and P1-2A (VP1 R188A), were processed by 3C^pro^ to yield VP0 and VP1 (Figs 5, 6 and S2 (supplementary material) even numbered lanes). In contrast, the P1-2A (VP1 Y185A) and P1-2A (VP1 R188A) mutants were highly resistant to cleavage by the 3C^pro^ (Fig 5, lanes 4 and 10). Furthermore, it was again apparent that the accumulation of these mutant P1-2A products in the cell lysates was lower than for the wt precursor and for the other mutants that could be processed (Fig 5, lanes 3 and 9). Thus, the single amino acid substitutions VP1 Y185A and VP1 R188A were individually able to severely inhibit processing at both the VP0/VP3 and the VP3/VP1 junctions within the P1-2A precursor and had a deleterious effect on the level of the unprocessed product generated within cells.

Surprisingly, none of the single alanine substitutions in the VP1 194–199 region had any effect on the processing of the junctions within the P1-2A precursor (S2 Fig, lanes 4, 6, 8, 10, 12 and 14). None of these produced the severe block on cleavage of the VP3-VP1 junction that was detected with the P1-2A (VP1 Δ194–199) mutant (Fig 4, lane 6). However, interestingly, the P1-2A (VP1 V193A) was processed more slowly at the VP3-VP1 junction compared to the P1-2A (wt) and the other alanine mutants (Fig 6, lane 12).

The cleavage of the VP1/2A junction of the P1-2A precursors with different alanine substitutions, was also investigated using the anti-2A antibody. As expected, the P1-2A (wt) generated a product of approximately 85 kDa corresponding to the P1-2A precursor in the absence of 3C^pro^ (see supplementary material S3 Fig, lane 1). However, in the presence of 3C^pro^, no product (containing 2A) was observed from the P1-2A (wt) showing that VP1/2A junction had been processed (S3 Fig, lane 2). The two mutants, P1-2A (VP1 C186A) and P1-2A (VP1 P187A) that were correctly processed at the VP0/VP3 and the VP3/VP1 junction also generated products corresponding to the P1-2A precursor in the absence of 3C^pro^ (S3 Fig, lanes 5 and 7). However, as with the wt protein, in the presence of 3C^pro^ no products including 2A could be detected, indicating that these two substitutions individually did not prevent processing at the VP1/2A junction. Neither of these mutant precursors, with single amino acid substitutions, which were highly resistant to cleavage at the VP0/VP3 and the VP3/VP1 junctions, i.e. P1-2A (VP1 Y185A) and P1-2A (VP1 R188A), could be detected by the anti-2A antibody, either in the absence or presence of 3C^pro^. Thus, we cannot conclude whether this junction was affected by these substitutions (S3 Fig, lanes 3, 4, 9 and 10). These results are consistent with the inability to detect the mutant capsid precursors P1-2A (VP1 Δ185–199), P1-2A (VP1 Δ185–189) and P1-2A (VP1 Δ188–192), with the anti-2A antibody, as shown in S1 Fig (see above).

### Influence of single amino acid substitutions within the YCPRP motif on virus infectivity

To confirm the importance of the YCPRP motif in the context of the virus itself, specific mutations have been introduced into the full-length FMDV cDNA, that encode single amino acid substitutions (to Ala) within the YCPRP motif. In addition, a deletion of the sequence encoding residues VP1 185–190 from the full-length FMDV cDNA was also made. RNA transcripts were prepared *in vitro* from each of the mutant plasmids and introduced into BHK cells. The initial harvests, prepared after 24h, were passaged onto fresh BHK cells and the appearance of cytopathic effect (CPE) observed. Clear CPE was observed with the wt transcript and from the mutants encoding the VP1 C186A and P189A substitutions. In contrast, no CPE was apparent for the mutants encoding the VP1 Y185A, P187A and R188A substitutions or with the mutant lacking residues VP1 185–190 (see Table 1). Sequencing of the P1-2A coding region from the rescued viruses (FMDV VP1 C186A and FMDV VP1 P189A revealed that the introduced mutations were retained and that no secondary mutations had occurred. These results verified the critical importance of residues Y185 and R188 in VP1 for P1-2A processing (Fig 5) and for virus infectivity. It is noteworthy that the P187A mutant was also non-infectious (Table 1) although the capsid precursor processing could be observed in the transient expression assay (see Fig 5, lane 8).

**Table 1:** Rescue of FMDVs from BHK cells.

RNA transcript	CPE at passage 1
FMDV (wt)	+++
FMDV (Δ185–190)	-
FMDV (Y185A)	-
FMDV (C186A)	+++
FMDV (P187A)	-
FMDV (R188A)	-
FMDV (P189A)	+++
No RNA	-

## Discussion

The FMDV 3C^pro^ is able to cleave a variety of different junction sequences in the virus polyprotein [33]. We have shown previously that blocking cleavage of one junction in the FMDV P1-2A did not affect processing of the other junctions [28]. In the current studies, it has been shown that modifications that modify or delete a short motif in the C-terminus of VP1, can prevent processing of the FMDV capsid precursor P1-2A at each of the usual cleavage sites, which are far separated, in the linear sequence, from the site of the modifications. The VP0/VP3 cleavage site is more than 400 amino acids away from the modified motif in the linear sequence while the VP3/VP1 junction is almost 200 amino acids away. It seems very likely that this reflects a major change in protein conformation for these mutant proteins. Viral proteins, like cellular proteins, are dependent on cellular chaperones for correct folding, assembly and function [24]. The viral capsid precursor must fold to a conformation that is soluble and recognizable by the viral protease to be processed. After the cleavage of the precursor, the mature capsid proteins assemble around the viral genome to form the protein shell, which contains 60 copies of each of the subunits. These structures must be stable both within, but also outside, the host cells to permit virus spread. Moreover, the virus particle must also be able to disassemble upon entry into cells to deliver the viral genome to initiate a new infection. Thus, the core structure of the capsid proteins (as distinct from the antigenic loops) is probably tightly constrained.

Within the picornavirus family, the general structure of the capsid proteins are very similar [2]. Several chaperones are known to facilitate folding of picornavirus capsid proteins [23,26]. The mature picornavirus capsid proteins are generated by cleavage of the P1, P1-2A or L-P1-2A precursors. Both Hsp90 and p23, a co-chaperone of Hsp90, have been reported to be required for processing of the PV P1 precursor into the mature structural proteins [23]. Similarly, inhibitors of Hsp90 have been shown to impede processing of the wt FMDV capsid precursor in cell-free assays [26]. However, interestingly, hepatitis A virus (HAV) is not sensitive to the inhibition of Hsp90 function [34]. This indicates that HAVs might employ other strategies for correct folding of the capsid precursor. However, it is noteworthy that HAV also has several unique characteristics that distinguish it from most other members of the picornavirus family, e.g. slow growth rate, lack of capsid protein myristoylation and use of only a single viral protease (3C^pro^) for polyprotein processing [35–38].

An earlier study showed that Hsp90 mediates PV P1 folding in cells. Inhibition of this chaperone lead to misfolding of P1, which resulted in the targeting of the PV P1 for degradation by the cellular quality-control system (proteasome pathway), and thus the level of the PV P1 was strongly reduced [23]. These observations are consistent with the results presented here on the FMDV P1-2A. All of the FMDV P1-2A precursors that cannot be processed by 3C^pro^ accumulated to a lower level than the P1-2A (wt). This was apparent for the truncated precursors (VP1 Y185Stop, VP1 L158Stop, VP1 A107Stop, VP1 L53Stop), precursors with small internal deletions (VP1 Δ185–199, VP1 Δ185–189, VP1 Δ182–192) and two precursors with single amino acid substitutions (VP1 Y185A and VP1 R188A). Thus, it may be that the mutant precursors, which cannot be processed, are misfolded and therefore targeted for degradation, hence the reduced level of these products within cells.

Interestingly, Geller et al., [23] showed that inhibition of the Hsp90 chaperone in a cell-free system (rabbit reticulocyte lysate), where the proteasomal degradation system is inhibited by free hemin, did not reduce the yield of P1 [23]. However, even in the absence of proteasomal degradation, the Hsp90 was still required for P1 to fold into a processing-competent conformation, since the PV P1 precursor, in the absence of Hsp90, adopted a misfolded conformation that could not be recognized by the 3CD^pro^ and thus could not be processed into the mature capsid proteins [23]. The clear resistance to processing of certain mutant FMDV P1-2A proteins (in which the YCPRP motif is modified or deleted) and their reduced accumulation within cells is entirely consistent with these results (see Fig 2B, Fig 3, Fig 4 and Fig 5).

**Fig 7:**
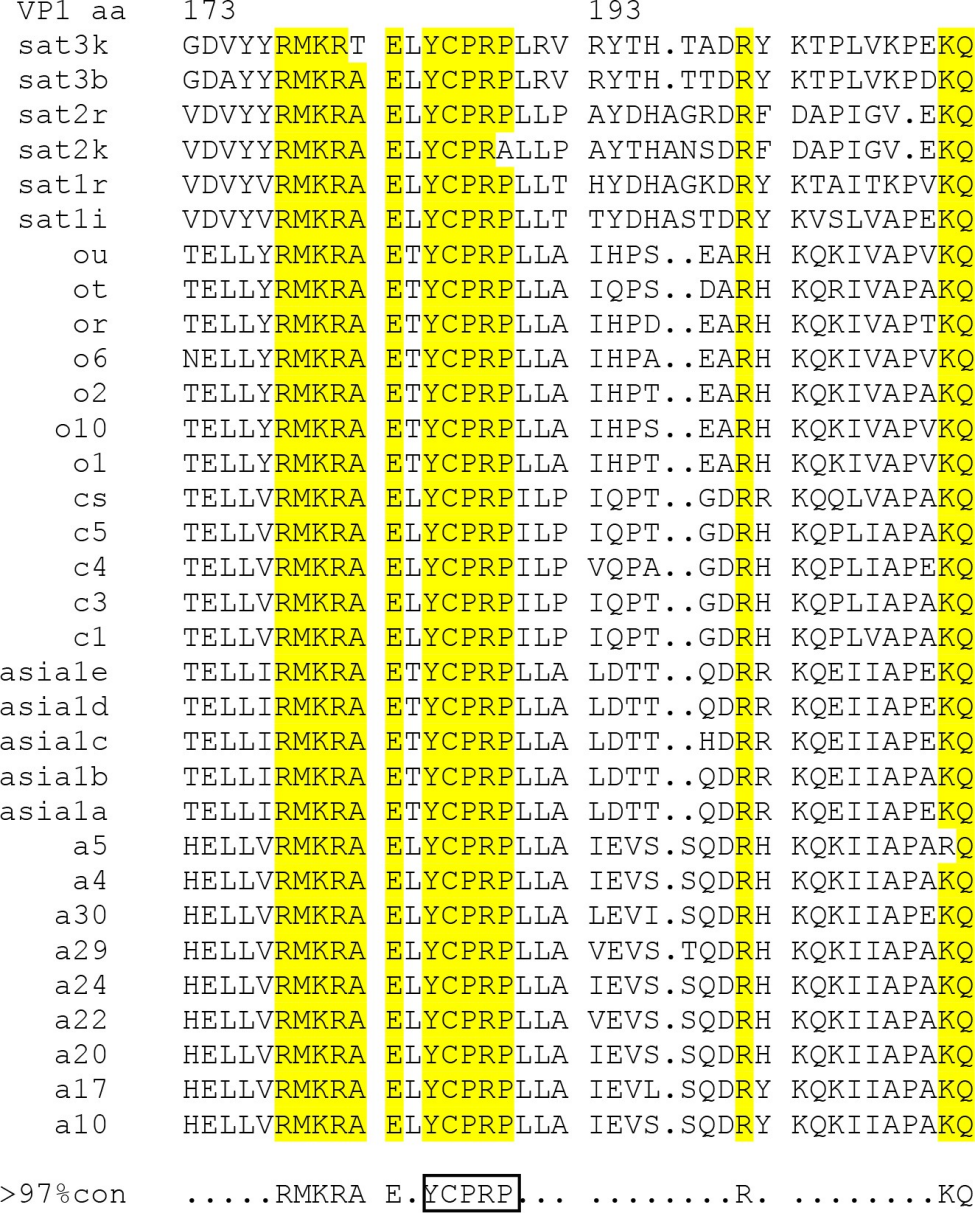
Alignment of the VP1 C-terminus from different FMDV serotypes Amino acid alignment of the VP1 C-terminus of different strains of FMDV, including examples of all seven serotypes. The yellow color represents amino acids that are more that 97% conserved among the different strains. The conserved motif YCPRP (VP1 185–189) is marked with a box in the consensus sequence. The alignment was prepared from sequences published previously [15].

**Fig 8:**
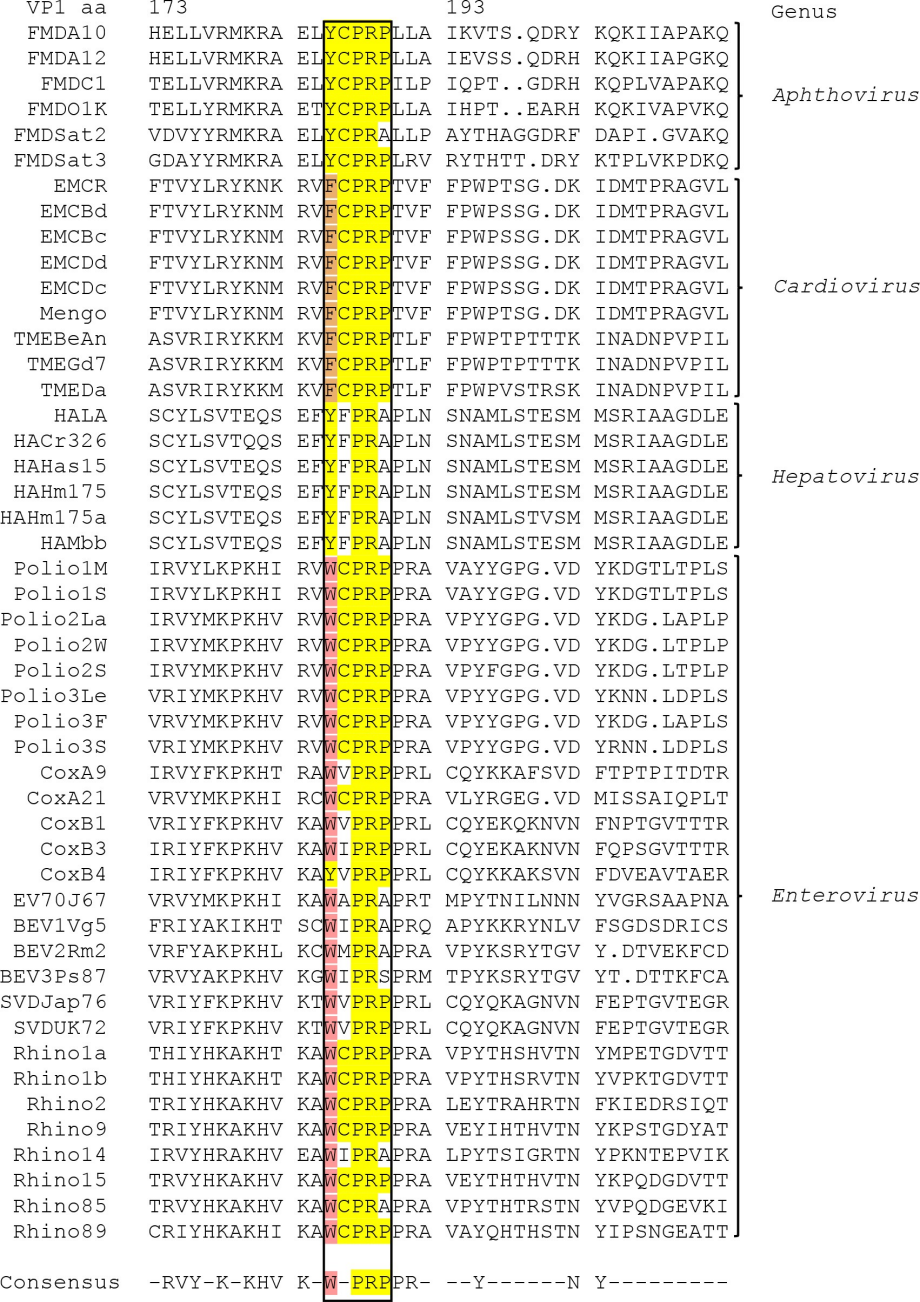
Alignment of different picornaviruses at the VP1 C-terminus Amino acid alignment of the VP1 C-terminus of diverse picornaviruses are shown. The black box indicates the highly conserved motif (VP1 185–189 in FMDV) that is important for correct processing of the capsid precursor. The sequences are numbered according to the FMDV residues. The yellow color represents amino acids that are identical with the YCPRP motif found in FMDV. Conservative substitutions are indicated with a similar color i.e. yellow for Y, orange for F and pink for W. The C residue at VP1 186 (according to FMDV VP1 numbers) is conserved in 31 out of 48 aligned picornaviruses (marked with yellow). The P and R at VP1 187 and 188 are 100% conserved amongst all of the aligned picornaviruses (marked with yellow). The P at VP1 189 (according to FMDV VP1 numbers) is conserved in 35 out of 48 of the aligned picornaviruses, differences from the P are seen in all of the hepatoviruses and a few of the enteroviruses. The alignment was taken from Ann Palmenberg’s laboratory website (http://www.virology.wisc.edu/acp/Aligns/aligns/picorna.p1) and highlighted manually. Abbreviations: FMDV = Foot-and-mouth disease virus, EMC = Encephalomyocarditis virus, TME = Theiler’s murine encephalomyelitis virus, HA = Hepatitis A virus, Cox = Coxsackievirus, EV = Enterovirus, BEV = Bovine enterovirus, SVD = Swine vesicular disease virus.

As indicated above, a critical region that is required for the correct processing of the FMDV capsid precursor has now been identified. This motif (YCPRP) is very highly conserved among FMDVs. Indeed, the YCPR sequence was found to be completely conserved in over 100 FMDV strains, with representatives from all 7 serotypes [15]); only variation to YCPRA has been observed (Fig 7). However, previously, no function for this conserved sequence had been identified. The YCPRP motif is also highly conserved among other picornaviruses as well, e.g. it exists as FCPRP in cardioviruses and WCPRP in enteroviruses, see Fig 8. Indeed, both Y to F and Y to W are very conservative amino acid substitutions, since all three amino acids have similar properties with non-polar, aromatic side chains. This high conservation likely reflects its importance for correct folding of the capsid precursor. The high resistance to cleavage of the junctions between the structural proteins following substitution of residues VP1 Y185 and VP1 R188 individually indicates that correct cleavage may be dependent on the interaction with several amino acids in this region and thus the whole motif seems to be of high importance for correct folding and subsequent processing of the capsid precursor. Furthermore, these results are consistent with the observations that the substitutions VP1 Y185A and VP1 R188A, that each prevent P1-2A processing by 3C^pro^ in cells (Fig 5), also block FMDV infectivity (Table 1). It is interesting to note that the VP1 P187A mutant was also non-infectious (Table 1) even though processing of the P1-2A could still be observed (Fig 5, lane 8). The high conservation of this motif clearly reflects its sensitivity to modification.

An earlier study has shown that removing 50 residues from the C-terminus of the PV VP1 prevented cleavage of the two junctions, VP0/VP3 and VP3/VP1, within the capsid precursor *in vitro* [30]. Significantly, these 50 amino acids include the highly conserved motif (WCPRP) identified here, and thus indicates the importance of this motif, not only for FMDV, but also more widely within the picornavirus family. Similarly, as indicated above, removal of 42 residues (including the YCPRP) from the C-terminus of the FMDV VP1 protein completely prevented cleavage of the capsid precursor by the 3C^pro^ in a cell-free system [29]. Recently, we have shown that blocking cleavage of one of the junctions within the FMDV P1-2A precursor did not block the cleavage of the other junction within the capsid precursor [28]. Thus, the severe inhibition of cleavage of both junctions likely reflects a changed overall structure of the capsid precursor, thereby preventing cleavage of both junctions.

**Fig 9:**
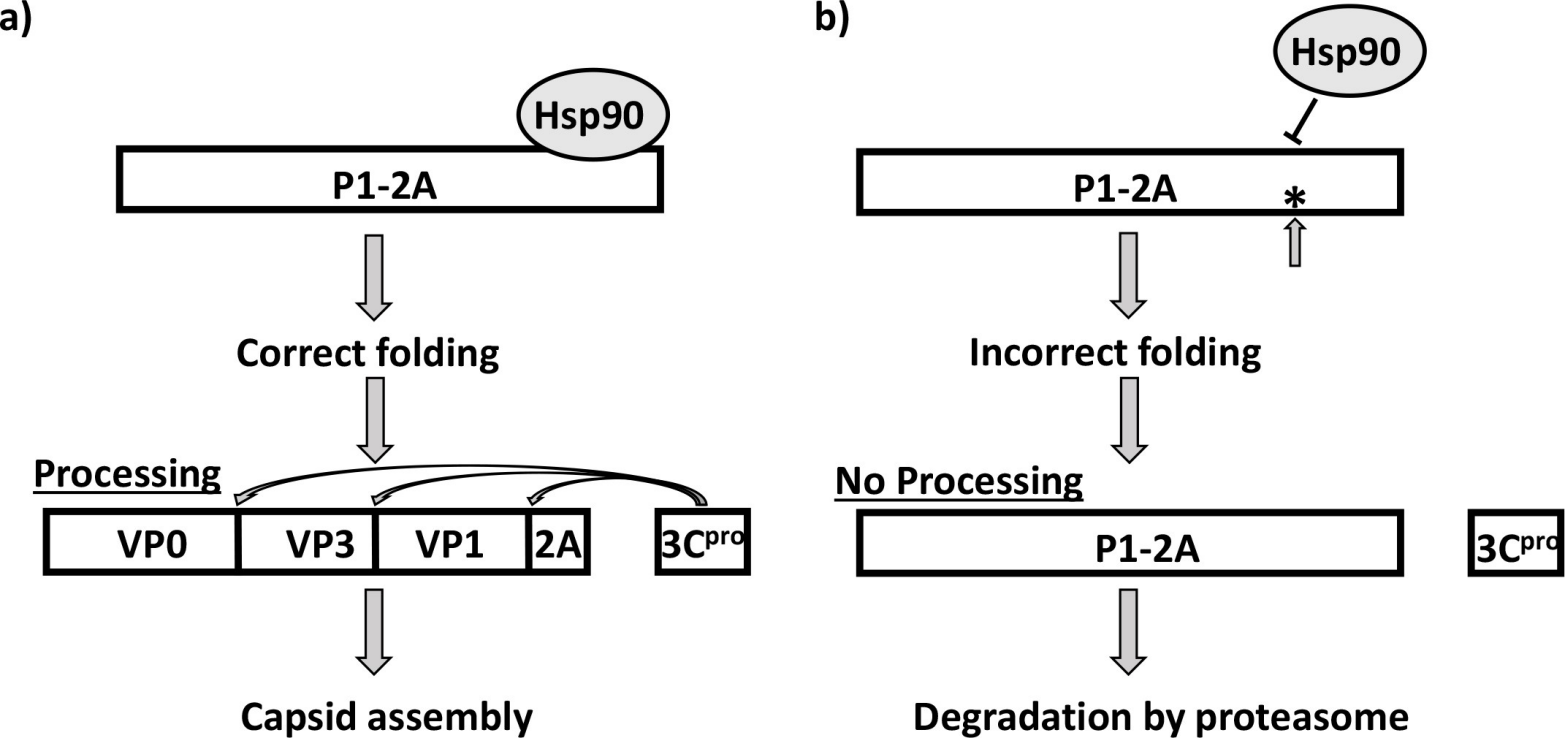
Model: Potential role of chaperone binding to a conserved motif for correct capsid precursor folding a) Hsp90 (or other chaperones and co-chaperones) is believed to bind to the P1-2A precursor (or P1 for enteroviruses) to facilitate folding into a state where the 3C^pro^ (or 3CD^pro^) is able to process the precursor to the mature structural capsid proteins prior to particle assembly. b) It is hypothesized that the conserved motif, YCPRP, in the C-terminus of VP1 is required for the binding of a chaperone (or co-chaperone). In the absence of this interaction, the precursor will be incorrectly folded and hence 3C^pro^ (or 3CD^pro^) will not recognize the cleavage sites; the misfolded, intact, precursor will be degraded by the proteasome.

It is interesting to note that in HAV, the equivalent region of VP1 has the sequence YFPRA, perhaps the two substitutions together account for the lack of sensitivity of HAV assembly to Hsp90 inhibitors [34]. It can be proposed that the conserved motif serves as a binding site for an important chaperone, e.g. Hsp90 (or its partners), that is necessary for correct protein folding. A proposed model for this interaction is shown in Fig 9. A co-chaperone of Hsp90, called p23, also seems to be involved in the correct folding of the PV P1. It has been reported that treatment with geldanamycin (GA) did not affect the PV P1-Hsp90 interaction, but abolished the P1-p23 interaction and thereby affected P1 maturation [23], thus indicating different possibilities for chaperone interaction at this specific site.

Picornaviruses are able to adapt very rapidly since they have an RNA dependent RNA polymerase with a high error rate and no error correction mechanism. However, Geller et al., [23] showed that PV was unable to adapt to an Hsp90-independent P1 folding pathway during several passages in cells in culture or in PV-infected mice when the function of Hsp90 was inhibited by the presence of GA. Thus it seems that for the virus to adapt to a folding pathway without the involvement of Hsp90 requires extensive change [23].

It is interesting that the deletion VP1 Δ194–199 strongly inhibited cleavage at the VP3-VP1 junction, without affecting the cleavage of the VP0-VP3 junction (Fig 4, lane 6). Surprisingly, the alanine scanning substitutions through this specific region did not identify any individual residue that affected cleavage of any of the junctions (S2 Fig, lanes 4, 6, 8, 10, 12 and 14). However, interestingly the P1-2A (VP1 V193A) mutant, modified at a residue adjacent to the deletion, also displayed a slower processing rate of this VP0/VP3 junction compared to the wt and the other alanine mutants (Fig 6, lane 12). However, this VP1 V193A mutant does not seem to affect the processing of the VP3-VP1 junction to the same extent as the VP1 Δ194–199 mutant. In addition, the VP1 Δ191–195 mutant also showed a lower processing of this VP3/VP1 junction (Fig 4B, lane 4) as judged by the elevated level of the VP3-VP1 product. These results indicate that the cleavage of the VP3-VP1 junction, may be dependent on the interaction with several amino acids and that residues within the VP1 aa 193–199 region are important for optimal processing of the VP3-VP1 junction, more than 190 aa away from the site.

We have noted previously that the K210E change in VP1 that severely limited processing at the VP1/2A junction also enhanced the yield of VP3-VP1-2A [14]. These studies also identified a genetic link between the processing of the VP1/2A junction and the substitution E83K in VP1. Furthermore, Escarmis et al., [39] showed that the substitution M54I within the VP1 of serotype C FMDV resulted in less efficient processing at the VP3/VP1 junction. Thus, there are multiple, complex, interactions, some of which operate “at a distance”, that govern picornavirus capsid protein processing and assembly.

## Methods

### Plasmid construction

The plasmid pO1K/A22 contains a T7 promoter upstream of a full-length FMDV cDNA with the A22 Iraq capsid coding sequence within an FMDV O1K backbone as previously described [28,40,41]. To investigate the effect of different modifications within the P1-2A, the FMDV cDNA was digested with *Apa*I and then religated to remove most of the sequence encoding the non-structural proteins (including the 3C^pro^) downstream of the 2A-peptide, as described previously [28]. These constructs contained a modified form (W52A substitution) of the L^pro^ to overcome the negative effect of the L protease on protein expression in cells. The modified L^pro^ with the W52A substitution retains the L/P1 cleavage activity but is defective at inducing cleavage of the translation initiation factor eIF4G [42]; the primer sequences used to make this modification are listed in S1 Table. This parental plasmid is referred to throughout as P1-2A (wt) and all modifications were made in this background.

### P1-2A plasmids specifying different truncations

Variants of the plasmid, with in-frame stop codons introduced to truncate the capsid precursor at different sites either at the start of the 2A sequence or within the VP1 coding sequence, were generated using site-directed mutagenesis [43]. Briefly, fragments were amplified in PCRs using Phusion High-Fidelity DNA Polymerase (Thermo Fisher Scientific), according to the manufacturer’s instructions, to create mega-primers, using the P1-2A (wt) plasmid as template and reverse primers specifying the introduction of STOP codons, together with the forward primer; 14TPN9_F, see S1 Table. The PCR products (between 350 and 900 bp in length depending on where the modification was made) were gel purified using the GeneJET Gel purification kit (Thermo Fisher Scientific). These PCR products were used as megaprimers for a second round of PCR (500 ng megaprimer, and 100 ng template), using the P1-2A (wt) as template to produce the modified plasmids. After the PCR and subsequent *Dpn*I digestion of the template plasmid, the products were transformed into chemically competent *Escherichia coli* (*E*. *coli*) cells. Plasmids were amplified from individual colonies, purified using the GeneJet Plasmid Miniprep Kit (Thermo Fisher Scientific) and screened by Sanger Sequencing using the BigDye Terminator v.3.1 Cycle Sequencing kit and a 3500 Genetic Analyzer (Applied Biosystems). Plasmids encoding the desired modifications were amplified and purified using the QIAGEN Plasmid Midi Kit (Qiagen). All of these constructs encoded P1 that was truncated at different sites by introducing two STOP codons. The plasmids (listed in S1 Table) were labelled to denote the location of the Stop codons as follows; P1-2A (2A L1Stop), P1-2A (VP1 I205Stop), P1-2A (VP1 D199Stop), P1-2A (VP1 Y185Stop), P1-2A (VP1 L158Stop), P1-2A (VP1 A107Stop) and P1-2A (VP1 L53Stop); for primer sequences, see S1 Table.

### Plasmids encoding P1-2A with internal deletions within the VP1 coding region

Variants of the P1-2A plasmid that express mutant proteins with various different deletions in the C-terminal region of VP1 between residues VP1 185 and VP1 199 were created using site-directed mutagenesis, essentially as described above; for primer sequences see S1 Table. The plasmids were labelled as follows: P1-2A (VP1 Δ185–199), P1-2A (VP1 Δ185–189), P1-2A (VP1 Δ188–192), P1-2A (VP1 Δ191–195) and P1-2A (VP1 Δ194–199). Furthermore, an additional positive control was included in which 13 amino acids within VP1 were deleted, this construct was called P1-2A (VP1 Δ142–154). An earlier study had shown that FMDV with this deletion is able to replicate [20,44].

### Alanine scanning mutagenesis of P1-2A

Alanine substitutions were introduced at the codons for each residue individually between VP1 185 and VP1 199, with the exception of VP1 A192, where the original alanine codon was substituted by one encoding a serine. The mutations were produced using site-directed mutagenesis, as described above; the primer sequences are listed in S2 Table. These modifications resulted in 15 different plasmids: P1-2A (VP1 Y185A), P1-2A (VP1 C186A), P1-2A (VP1 P187A), P1-2A (VP1 R188A), P1-2A (VP1 P189A), P1-2A (VP1 L190A), P1-2A (VP1 L191A), P1-2A (VP1 A192S), P1-2A (VP1 V193A), P1-2A (VP1 E194A), P1-2A (VP1 V195A), P1-2A (VP1 S196A), P1-2A (VP1 S197A), P1-2A (VP1 Q198A) and P1-2A (VP1 D199A).

### Transient expression assays

Baby hamster kidney (BHK) cells (originally obtained from the ATCC (CCL-10)) were grown in 35-mm wells to about 90% confluence, when they were infected with the recombinant vaccinia virus, termed vTF7-3 [45] that expresses the T7 RNA polymerase. All the various P1-2A plasmids and the 3C plasmid (pSKRH3C [46]) express the FMDV cDNA under the control of a T7 promotor. After one hour incubation at 37°C, the vaccinia virus was removed and the cells were transfected with the specified plasmid DNA using FuGENE 6 (Promega), as described previously [47]. To obtain the highest levels of processed capsid protein expression, 1000 ng of the P1-2A plasmid alone or with 10 ng of the 3C^pro^ plasmid were used for each transfection of cells [28]. The cells were incubated in a CO_2_ incubator, at 37°C overnight and then, after removal of the medium, lysed with 500 μl Buffer C (20mM Tris-HCl (pH 8.0), 125 mM NaCl and 0.5% NP-40); the cell extracts were clarified by centrifugation at 18,000 x g for 10 min at 4°C.

### Immunoblot analysis

Immunoblotting was performed using clarified cell lysates mixed with 2 x Laemmli sample buffer (Bio-Rad) (containing 25 mM DTT). The proteins were separated by SDS-PAGE using a 12% Bis-Tris gels (Bio-Rad) and transferred to PVDF membranes (Milipore), by wet blotting, at 200 mA for 1.5 hours. PBS containing bovine serum albumin (BSA) (5%) and Tween20 (0.1%) was used as blocking buffer (1 hour at room temperature (RT)) and dilution buffer for the guinea pig anti-FMDV O-Manisa (Man) antisera (prepared “in house”, as used previously [28]) (overnight at 4°C) and their corresponding secondary antibodies (2 hours at RT). Guinea pig anti-FMDV O-Man antisera was used for detection, since it is very efficient in detecting the denatured capsid proteins from various FMDV serotypes. PBS containing skimmed milk powder (5%) and Tween20 (0.1%) was used as blocking buffer (1 hour at RT) and dilution buffer for the primary guinea pig anti-FMDV A-Iraq antisera (prepared “in house”, as used previously [28]) and the anti-2A-peptide antibody (overnight at 4°C) and their corresponding secondary antibody (2 hours at RT). The proteins were detected using the following primary antibodies: guinea pig anti-FMDV O-Man antisera (1:1000), guinea pig anti-FMDV A-Iraq antisera (1:500) or FMDV anti-2A-peptide antibody (1:1000) (Rabbit, ABS31 Merck Millipore). Appropriate HRP-conjugated secondary antibodies (Dako) and a chemiluminescence detection kit (Pierce ECL Western Blotting Substrate, Thermo Fisher Scientific) were used to detect the proteins bound by the primary antibodies. Images were captured using a Chem-Doc XRS system (Bio-Rad).

### Virus modification and rescue

The plasmid pO1K/A22 contains a full-length cDNA corresponding to a chimeric FMDV genome as previously described [28]. It includes the capsid coding sequence from FMDV A22 Iraq and the rest of the genome from FMDV O1K. Briefly, fragments were amplified in PCRs, using the pO1K/A22 plasmid as template, together with primers specifying the desired mutations, see S1 and S2 Tables. The PCR products were gel purified and used as megaprimers for a second round of PCR, again using the wt pO1K/A22 as template to make full-length plasmids of approximately 11,000 bp. After the PCR and subsequent *Dpn*I digestion of the template DNA, the products were introduced into *E*. *coli* cells. Plasmids were amplified from individual colonies and sequenced. The plasmids, containing the full-length wt or mutant FMDV cDNAs, were linearized by digestion with *Hpa*I and then transcribed *in vitro* using the MEGAscript T7 Transcription Kit (Thermo Fisher Scientific). An aliquot (1 μL) of each RNA sample was visualized following agarose gel electrophoresis to check yield and integrity and the rest (19 μL) was introduced into BHK cells by electroporation as described previously [28]. The cells were transferred to Falcon flasks and Eagle’s medium containing 5% calf serum was added. The cells were incubated overnight at 37°C and then harvested. Aliquots (1ml) of the harvest were inoculated onto fresh BHK cells and the appearance of cytopathic effect monitored at 1 and 2 days post-inoculation. For samples displaying CPE, viral RNA was isolated using the RNeasy Mini Kit (Qiagen) and reverse transcribed using Ready-To-Go You-Prime First-Strand Beads (GE Healthcare Life Sciences) together with random primers. The cDNA corresponding to the P1-2A region was amplified as four overlapping fragments of around 1000 bp by AmpliTaq Gold DNA Polymerase (Thermo Fisher Scientific) as described previously [28] and then sequenced. For each RNA, a negative control, lacking the reverse transcriptase, was included in the RT-PCRs to verify that the PCR products were obtained from viral RNA and not from residual plasmid template.

## Supporting information

S1 Fig Effect of small deletions on processing of P1-2A at the FMDV VP1/2A junction. The P1-2A precursor (wt or with small deletions as indicated) was expressed alone or with 3C^pro^ in transient expression assays in BHK cells as for Fig 3. Cell lysates were prepared and analyzed by immunoblotting using rabbit anti-2A antibodies. Bound antibodies were visualized using the anti-rabbit HRP-conjugated secondary antibodies and chemiluminescence detection. The odd numbered lanes show the P1-2A precursors expressed alone and the even numbered lanes show the precursor co-expressed with 3C^pro^. Molecular mass markers (kDa) are indicated on the left. A negative control (No DNA) is included in lane 15. A positive control with a deletion known to be tolerated in replicating FMDV [20] was included, P1-2A (VP1 Δ142–154), lanes 13 and 14. Note, the free 2A peptide (18 residues long) is too small to detect in this system.
(TIF)

S2 Fig Multiple residues within the VP1 194–199 region affect processing of the VP3/VP1 junction. The P1-2A precursors (wt or with alanine substitutions between VP1 194 and VP1 199, as indicated) were expressed alone or in the presence of 3C^pro^ and analyzed by immunoblotting. The different capsid proteins are indicated on the right of the figure. Proteins were detected using guinea pig anti-FMDV O-Man antisera (a) or guinea pig anti-FMDV A-Iraq antisera (b). Bound antibodies were visualized using the anti-guinea pig HRP-conjugated secondary antibodies and a chemiluminescence detection kit. Molecular mass markers (kDa) are indicated on the left. A negative control (No DNA) is included in lane 15.
(TIF)

S3 Fig Analysis of processing at the FMDV VP1/2A junction in P1-2A precursors modified at individual residues between VP1 185 and 188. The P1-2A precursors (wt or having alanine substitutions between VP1 185 and VP1 188) were expressed alone or in the presence of 3C^pro^ and analyzed by immunoblotting. The P1-2A was detected using rabbit anti-2A antibodies and visualized using rabbit HRP-conjugated secondary antibodies and a chemiluminescence detection kit. Molecular mass markers (kDa) are indicated on the left. A negative control (No DNA) is included in lane 11. Note, the free 2A peptide (18 residues long) is too small to detect in this system.
(TIF)

S1 Table Primers used for site-directed mutagenesis. (DOCX)

S2 Table Primers used for alanine scanning mutagenesis. (DOCX)
