# Supplementary figures and images for: Identification of a short, highly conserved, motif required for picornavirus capsid precursor processing at distal sites
Source: PLoS Pathog. 2019 Jan 18;15(1):e1007509. doi: 10.1371/journal.ppat.1007509 (PMC6338358; doi:10.1371/journal.ppat.1007509)

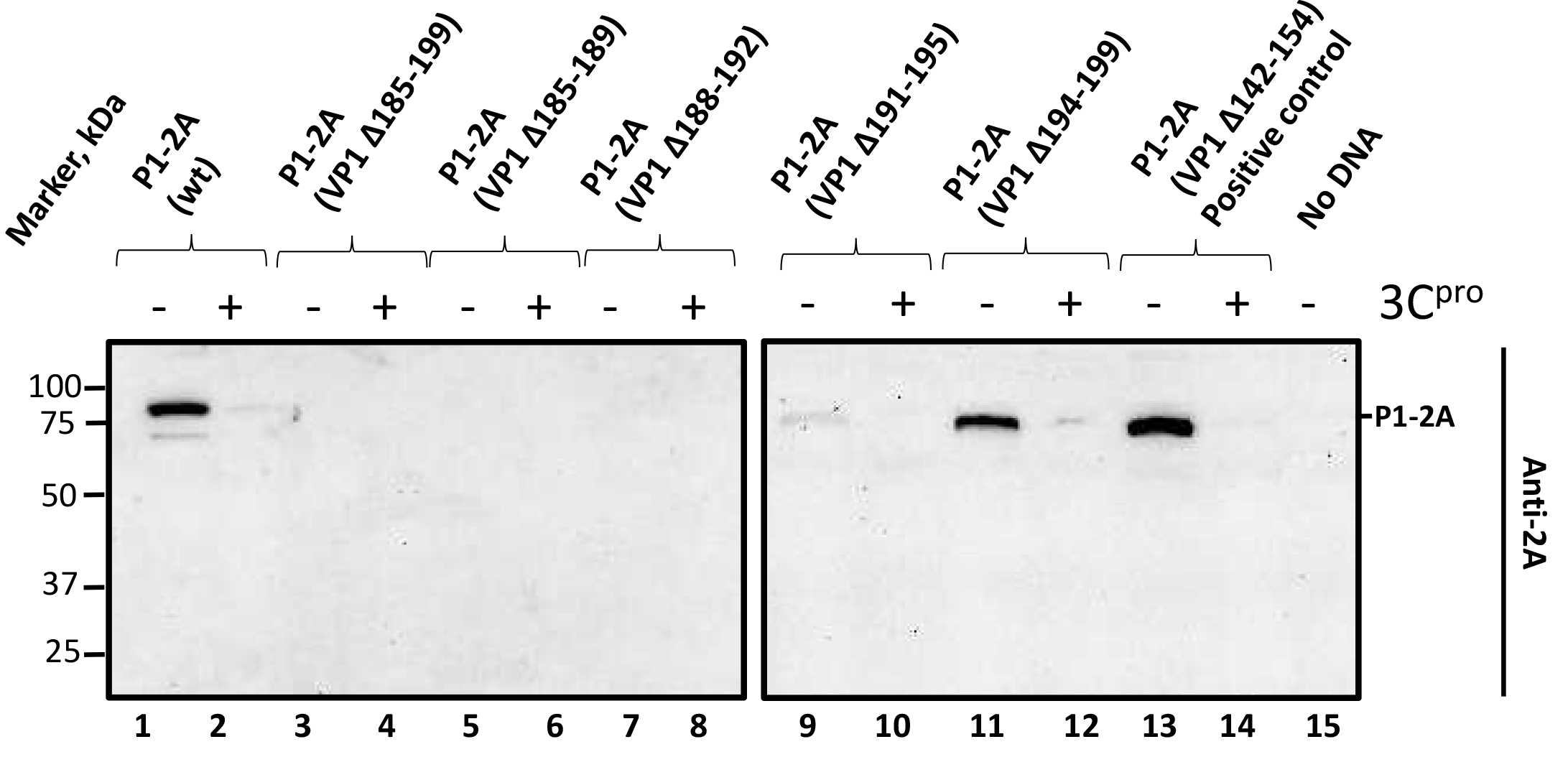

Supplement: S1 Fig — The P1-2A precursor (wt or with small deletions as indicated) was expressed alone or with 3Cpro in transient expression assays in BHK cells as for Fig 3. Cell lysates were prepared and analyzed by immunoblotting using rabbit anti-2A antibodies. Bound antibodies were visualized using the anti-rabbit HRP-conjugated secondary antibodies and chemiluminescence detection. The odd numbered lanes show the P1-2A precursors expressed alone and the even numbered lanes show the precursor co-expressed with 3Cpro. Molecular mass markers (kDa) are indicated on the left. A negative control (No DNA) is included in lane 15. A positive control with a deletion known to be tolerated in replicating FMDV [20] was included, P1-2A (VP1 Δ142–154), lanes 13 and 14. Note, the free 2A peptide (18 residues long) is too small to detect in this system. (TIF) [file ppat.1007509.s001.tif]

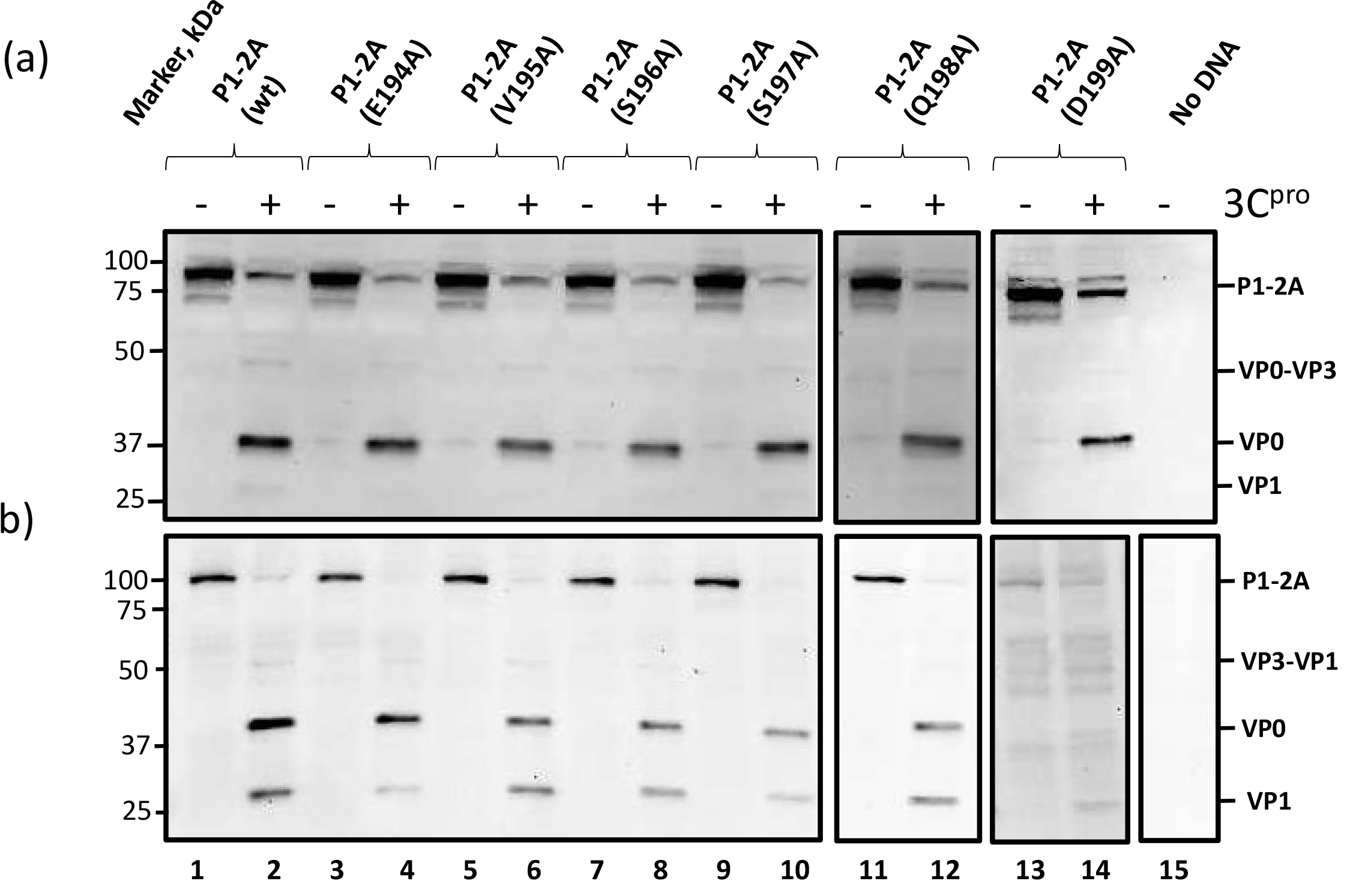

Supplement: S2 Fig — The P1-2A precursors (wt or with alanine substitutions between VP1 194 and VP1 199, as indicated) were expressed alone or in the presence of 3Cpro and analyzed by immunoblotting. The different capsid proteins are indicated on the right of the figure. Proteins were detected using guinea pig anti-FMDV O-Man antisera (a) or guinea pig anti-FMDV A-Iraq antisera (b). Bound antibodies were visualized using the anti-guinea pig HRP-conjugated secondary antibodies and a chemiluminescence detection kit. Molecular mass markers (kDa) are indicated on the left. A negative control (No DNA) is included in lane 15. (TIF) [file ppat.1007509.s002.tif]

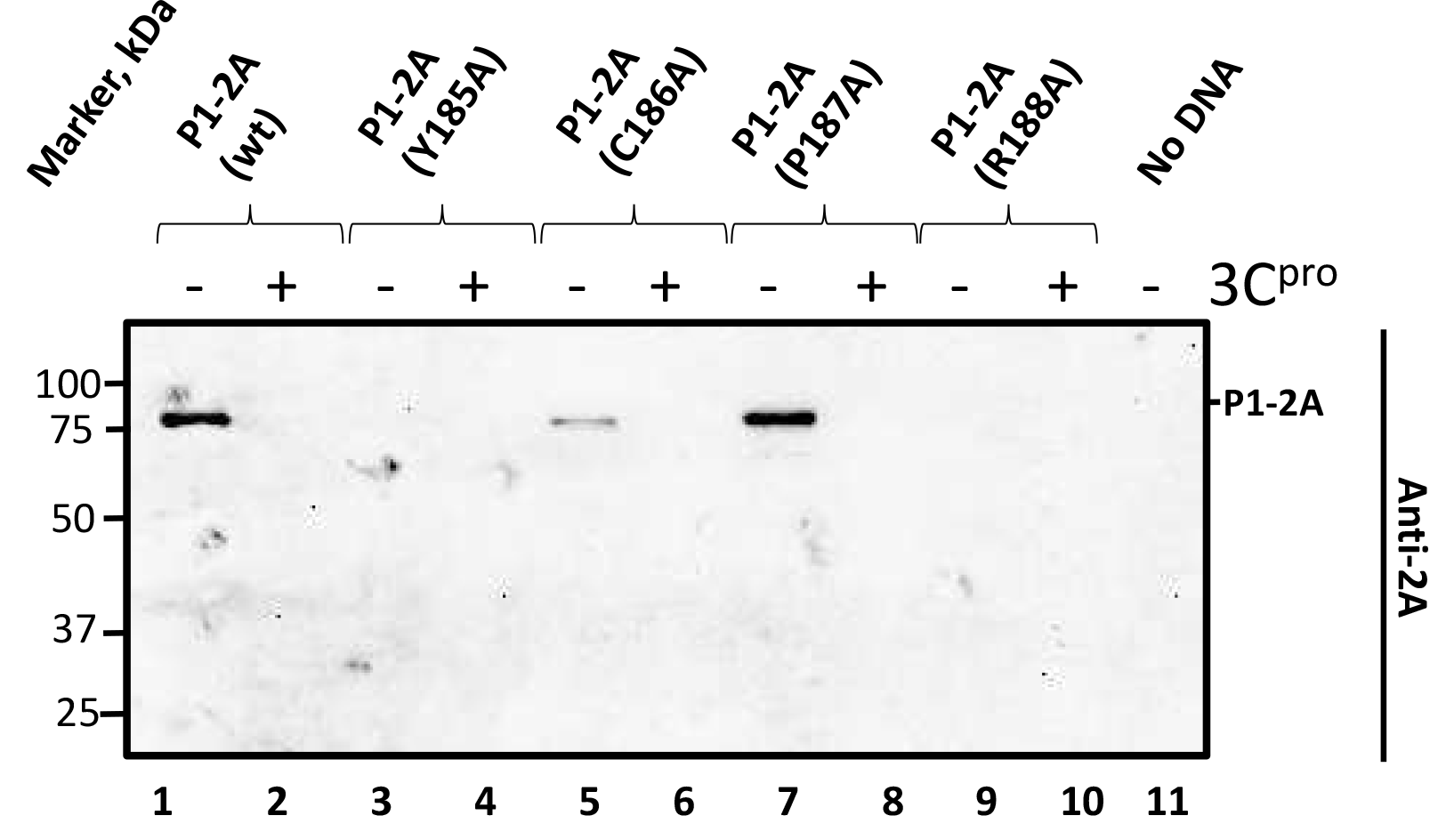

Supplement: S3 Fig — The P1-2A precursors (wt or having alanine substitutions between VP1 185 and VP1 188) were expressed alone or in the presence of 3Cpro and analyzed by immunoblotting. The P1-2A was detected using rabbit anti-2A antibodies and visualized using rabbit HRP-conjugated secondary antibodies and a chemiluminescence detection kit. Molecular mass markers (kDa) are indicated on the left. A negative control (No DNA) is included in lane 11. Note, the free 2A peptide (18 residues long) is too small to detect in this system. (TIF) [file ppat.1007509.s003.tif]
