# Supplementary material for: Identification of a short, highly conserved, motif required for picornavirus capsid precursor processing at distal sites
Source: PLoS Pathog. 2019 Jan 18;15(1):e1007509. doi: 10.1371/journal.ppat.1007509 (PMC6338358; doi:10.1371/journal.ppat.1007509)
Supplement: S1 Table — (DOCX) [file ppat.1007509.s004.docx]

**Table S1: Primers used for site-directed mutagenesis**

| Primers for Megaprimers | Changes | Name |
| --- | --- | --- |
| **Plasmids for the Transient expression assay, introducing Leader (W52A) substitution** | | |
| SG31_Fw: CCACGACAACTGCgcGTTGAACGCC  14TPN6_Rev: CAAACAGGTGCTTCTTGAAAAATCTTTC | Leader (W52A) TGG->GCG | P1-2A (wt) |
| **Plasmids for the Transient expression assay, introducing Stop codons (truncations) in VP1, note that all constructs contains the Leader (W52A) substitution** | | |
| 14TPN9_Fw: ATCCATGCTGAGTGGGACACAG  1PTK41_R: GAGCAAATCGAAGTTttattaTTGTTTTGC | 2A L1StopStop  (CTTTTG-> TAATAA) | P1-2A (2A L1Stop) |
| 14TPN9_Fw: ATCCATGCTGAGTGGGACACAG  1PTK40_R: AAGTTGTTTTGCAGGttattaGATCTTCTG | VP1 I205StopStop  (ATTGCA -> TAATAA) | P1-2A (VP1 I205Stop) |
| 14TPN9_Fw: ATCCATGCTGAGTGGGACACAG  1PTK39_R: ATCTTCTGTTTGTGttattaTTGAGACGAC | VP1 D199StopStop  (GACAGA -> TAATAA) | P1-2A (VP1 D199Stop) |
| 14TPN9_Fw: ATCCATGCTGAGTGGGACACAG  1PTK38_R: CAACAGTGGTCTGGGttattaGAGTTCGGC | VP1 Y185StopStop  (TACTGC -> TAATAA) | P1-2A (VP1 Y185Stop) |
| 14TPN9_Fw: ATCCATGCTGAGTGGGACACAG  1PTK37_R: AAGTTGAAAGAAGCttattaCTGAGCGGCG | VP1 L158StopStop  (CTTCCT -> TAATAA) | P1-2A (VP1 L158Stop) |
| 14TPN9_Fw: ATCCATGCTGAGTGGGACACAG  1PTK36_R: AATGGTGCCTTGAGttattaGGTGGGGTTG | VP1 A107StopStop  (GCCTAC -> TAATAA) | P1-2A (VP1 A107Stop) |
| 14TPN9_Fw: ATCCATGCTGAGTGGGACACAG  1PTK35_R: TGTTGGTGGGTTTGttattaGTCAATGACA | VP1 L53StopStop  (CTCATG -> TAATAA) | P1-2A (VP1 L53Stop) |
| **Plasmids for the Transient expression assay, introducing small deletions in the VP1 C-terminus between VP1 185 and VP1 199 , note that all constructs contains the Leader (W52A) substitution** | | |
| 14TPN9_Fw: ATCCATGCTGAGTGGGACACAG  1PTK42_R: TGATCTTCTGTTTGTGTC**TG**AGTTCGGCACGC |  | **P1-2A** **(VP1 Δ185-199)** |
| 14TPN9_Fw: ATCCATGCTGAGTGGGACACAG  1PTK43_R: ACACCTCCACTGCCAACA**GG**AGTTCGGCACGCT |  | **P1-2A (VP1 Δ185-189)** |
| 14TPN9_Fw: ATCCATGCTGAGTGGGACACAG  1PTK44_R: TTGAGACGACACCTCCA**CG**GGGCAGTAGAGT |  | **P1-2A** **(VP1 Δ188-192)** |
| 14TPN9_Fw: ATCCATGCTGAGTGGGACACAG  1PTK45_R: TTGTGTCTGTCTTGAGACG**AC**AGTGGTCTGG |  | **P1-2A (VP1 Δ191-195)** |
| 14TPN9_Fw: ATCCATGCTGAGTGGGACACAG  1PTK46_R: ATGATCTTCTGTTTGTGTC**TC**TCCACTGCCAA |  | **P1-2A** **(VP1 Δ194-199)** |
| 14TPN9_Fw: ATCCATGCTGAGTGGGACACAG  1PTK47_R: AGCAGGAAGCTGAGCGG**CC**GTACCACCTGCGGA |  | **P1-2A** **(VP1 Δ142-154)** **Positive control** |

Small letters = Nucleotide changes. Bold = Deletion between the two bold nucleotides.
