# Supplementary material for: Identification of a short, highly conserved, motif required for picornavirus capsid precursor processing at distal sites
Source: PLoS Pathog. 2019 Jan 18;15(1):e1007509. doi: 10.1371/journal.ppat.1007509 (PMC6338358; doi:10.1371/journal.ppat.1007509)
Supplement: S2 Table — (DOCX) [file ppat.1007509.s005.docx]

**Table S2: Primers used for alanine-scanning mutagenesis**

| Primers for Megaprimers | Changes | Name |
| --- | --- | --- |
| **Plasmids for the Transient expression assay, introducing alanine substitutions in the VP1 C-terminus between VP1 185 and VP1 199 (with the exception of VP1 192 where an alanine was substituted to a serine, note that all constructs contains the Leader (W52A) substitution** | | |
| 14TPN9_Fw: ATCCATGCTGAGTGGGACACAG  1PTK48_R: CAGTGGTCTGGGGCAagcGAGTTCGGCA | VP1 Y185A  (TAC-> AGC) | P1-2A (VP1 Y185A) |
| 14TPN9_Fw: ATCCATGCTGAGTGGGACACAG  1PTK49_R: CCAACAGTGGTCTGGGagcGTAGAGTTC | VP1 C186A  (TGC-> AGC) | P1-2A (VP1 C186A) |
| 14TPN9_Fw: ATCCATGCTGAGTGGGACACAG  1PTK50_R: ACTGCCAACAGTGGTCTagcGCAGTAGA | VP1 P187A  (CCC-> AGC) | P1-2A (VP1 P187A) |
| 14TPN9_Fw: ATCCATGCTGAGTGGGACACAG  1PTK51_R: CCACTGCCAACAGTGGagcGGGGCAGTA | VP1 R188A  (AGA-> AGC) | P1-2A (VP1 R188A) |
| 14TPN9_Fw: ATCCATGCTGAGTGGGACACAG  1PTK52_R: CCTCCACTGCCAACAGagcTCTGGGGCA | VP1 P189A  (CCA-> AGC) | P1-2A (VP1 P189A) |
| 14TPN9_Fw: ATCCATGCTGAGTGGGACACAG  1PTK53_R: ACACCTCCACTGCCAAagcTGGTCTGGG | VP1 L190A  (CTG-> AGC) | P1-2A (VP1 L190A) |
| 14TPN9_Fw: ATCCATGCTGAGTGGGACACAG  1PTK54_R: CGACACCTCCACTGCagcCAGTGGTCTG | VP1 L191A  (TGG-> AGC) | P1-2A (VP1 L191A) |
| 14TPN9_Fw: ATCCATGCTGAGTGGGACACAG  1PTK55_R: TGAGACGACACCTCCACagaCAACAGTG | VP1 A192S  (GCA-> TCT) | P1-2A (VP1 A192S) |
| 14TPN9_Fw: ATCCATGCTGAGTGGGACACAG  1PTK56_R: CTTGAGACGACACCTCagcTGCCAACAG | VP1 V193A  (GTG-> AGC) | P1-2A (VP1 V193A) |
| 14TPN9_Fw: ATCCATGCTGAGTGGGACACAG  1PTK57_R: GTCTTGAGACGACACagcCACTGCCAAC | VP1 E194A  (GAG-> AGC) | P1-2A (VP1 E194A) |
| 14TPN9_Fw: ATCCATGCTGAGTGGGACACAG  1PTK58_R: TGTCTGTCTTGAGACGAagcCTCCACTG | VP1 V195A  (GTG-> AGC) | P1-2A (VP1 V195A) |
| 14TPN9_Fw: ATCCATGCTGAGTGGGACACAG  1PTK59_R: TTTGTGTCTGTCTTGAGAagcCACCTCC | VP1 S196A  (TCG-> AGC) | P1-2A (VP1 S196A) |
| 14TPN9_Fw: ATCCATGCTGAGTGGGACACAG  1PTK60_R: CTGTTTGTGTCTGTCTTGagcCGACACC | VP1 S197A  (TCT-> AGC) | P1-2A (VP1 S197A) |
| 14TPN9_Fw: ATCCATGCTGAGTGGGACACAG  1PTK61_R: TCTGTTTGTGTCTGTCagcAGACGACAC | VP1 Q198A  (CAA-> AGC) | P1-2A (VP1 Q198A) |
| 14TPN9_Fw: ATCCATGCTGAGTGGGACACAG  1PTK62_R: TCTGTTTGTGTCTagcTTGAGACGACAC | VP1 D199A  (GAC-> AGC) | P1-2A (VP1 D199A) |

Small letters = Nucleotide changes.
